# Accumulation of amyloid precursor protein C-terminal fragments triggers mitochondrial structure, function, and mitophagy defects in Alzheimer’s disease models and human brains

**DOI:** 10.1007/s00401-020-02234-7

**Published:** 2020-10-20

**Authors:** Loan Vaillant-Beuchot, Arnaud Mary, Raphaëlle Pardossi-Piquard, Alexandre Bourgeois, Inger Lauritzen, Fanny Eysert, Paula Fernanda Kinoshita, Julie Cazareth, Céline Badot, Konstantina Fragaki, Renaud Bussiere, Cécile Martin, Rosanna Mary, Charlotte Bauer, Sophie Pagnotta, Véronique Paquis-Flucklinger, Valérie Buée-Scherrer, Luc Buée, Sandra Lacas-Gervais, Frédéric Checler, Mounia Chami

**Affiliations:** 1 Institut of Molecular and Cellular Pharmacology, Laboratory of Excellence DistALZ, Université Côte d’Azur, INSERM, CNRS, Sophia-Antipolis, 06560 Valbonne, France; 2 Department of Pharmacology, Instituto de Ciências Biomédicas, Universidade de São Paulo, São Paulo, Brazil; 3 Université Côte d’Azur, CHU, Inserm, CNRS, IRCAN, Nice, France; 4 Department of Medicine, Burlington Danes Building, Hammersmith Hospital Campus, Imperial College London, UK Dementia Research Institute, Du Cane Road, London, W12 0NN UK; 5 Université Côte d’Azur, Centre Commun de Microscopie Appliquée (CCMA), Parc Valrose, 06108 Nice, France; 6 Univ. Lille, Inserm, CHU-Lille, Lille Neuroscience and Cognition, Place de Verdun, 59045 Lille, France; 7 Inserm UMR-S 1172, Laboratory of Excellence DistALZ, ‘Alzheimer and Tauopathies’, Bâtiment Biserte, rue Polonovski, 59045 Lille Cedex, France

**Keywords:** Alzheimer’s disease, Amyloid precursor protein, Amyloid beta, APP-CTFs, C99, C83, Mitochondria, Mitophagy

## Abstract

Several lines of recent evidence indicate that the amyloid precursor protein-derived C-terminal fragments (APP-CTFs) could correspond to an etiological trigger of Alzheimer’s disease (AD) pathology. Altered mitochondrial homeostasis is considered an early event in AD development. However, the specific contribution of APP-CTFs to mitochondrial structure, function, and mitophagy defects remains to be established. Here, we demonstrate in neuroblastoma SH-SY5Y cells expressing either APP Swedish mutations, or the β-secretase-derived APP-CTF fragment (C99) combined with β- and γ-secretase inhibition, that APP-CTFs accumulation independently of Aβ triggers excessive mitochondrial morphology alteration (i.e., size alteration and cristae disorganization) associated with enhanced mitochondrial reactive oxygen species production. APP-CTFs accumulation also elicit basal mitophagy failure illustrated by enhanced conversion of LC3, accumulation of LC3-I and/or LC3-II, non-degradation of SQSTM1/p62, inconsistent Parkin and PINK1 recruitment to mitochondria, enhanced levels of membrane and matrix mitochondrial proteins, and deficient fusion of mitochondria with lysosomes. We confirm the contribution of APP-CTFs accumulation to morphological mitochondria alteration and impaired basal mitophagy in vivo in young 3xTgAD transgenic mice treated with γ-secretase inhibitor as well as in adeno-associated-virus-C99 injected mice. Comparison of aged 2xTgAD and 3xTgAD mice indicates that, besides APP-CTFs, an additional contribution of Aβ to late-stage mitophagy activation occurs. Importantly, we report on mitochondrial accumulation of APP-CTFs in human post-mortem sporadic AD brains correlating with mitophagy failure molecular signature. Since defective mitochondria homeostasis plays a pivotal role in AD pathogenesis, targeting mitochondrial dysfunctions and/or mitophagy by counteracting early APP-CTFs accumulation may represent relevant therapeutic interventions in AD.

**Electronic supplementary material:**

The online version of this article (10.1007/s00401-020-02234-7) contains supplementary material, which is available to authorized users.

## Introduction

Neurons, as post-mitotic and high-energy demanding cells, are especially sensitive to impairment of mitochondria functions and defective quality control processes. Mitochondria provide most of the cell energetic demands by generating ATP, the product of the oxidative phosphorylation (OXPHOS). Along with cell life control, mitochondria are also implicated in harmful reactive oxygen species (ROS) production and apoptotic cell death [57]. Mitochondria homeostasis is governed by equilibrium between genesis and clearance by mitophagy, the selective degradation process of defective or functionally altered mitochondria [64]. One major mechanism of mitophagy is the PTEN (Phosphatase and TENsin homolog)-induced putative kinase 1 (PINK1)/Parkin pathway. Unhealthy mitochondria with a loss of membrane potential (ΔΨm) stabilize PINK1 at the outer mitochondrial membrane. PINK1 recruits the E3 Ubiquitin ligase Parkin to generate polyubiquitinated proteins recognized by mitophagy adapters (i.e., p62/SQSTM1). Flagged mitochondria are recruited by lipid-bound microtubule-associated protein 1A/1B-light chain 3 (LC3-II) present on the double-membrane called pre-autophagosome structure. Sequestered dysfunctional mitochondria in the mitophagosome are ultimately delivered to lysosomes for degradation [64]. Perturbations of mitochondria physiology are considered hallmarks of several neurodegenerative disorders [28], including Alzheimer’s disease (AD) [34].

AD is a progressive neurodegenerative pathology leading to dementia characterized by the accumulation in AD-affected brains of hyperphosphorylated Tau (pTau) protein and of toxic amyloid beta (Aβ) peptide, a product of amyloid precursor protein (APP) processing [19]. The processing of APP generates several fragments with partially documented physiopathological functions [23, 55]. APP C-terminal fragments (APP-CTFs) are produced through the cleavage of APP by β- and α-secretases generating C99 (99-amino acids) and C83 (83-amino acids), respectively. Subsequent cleavages of C99 and C83 by γ-secretase generate Aβ and p3, respectively, and the APP intracellular domain (AICD). Noticeably, C99 also undergoes cleavage by α-secretase producing C83 [14, 29].

Most of AD-related mitochondrial dysfunctions including decreased ATP levels, impaired OXPHOS activity, disrupted mitochondrial membrane potential (ΔΨm), and increased levels of ROS were described to be Aβ-dependent in in vitro and in vivo AD study models [2, 68, 80]. Moreover, recent studies reported defective mitophagy linked to Aβ and pTau [17, 35, 50, 69]. However, the specific role of APP-CTFs, and more particularly its deleterious accumulation [44], in mitochondrial structure, function, and mitophagy dysfunctions in AD remain to be deciphered. Strikingly, we and others recently demonstrated the localization of APP-CTFs in the subcellular microdomain between the endoplasmic reticulum (ER) and mitochondria [20, 63], thus altering MAMs and mitochondrial membranes lipid composition [4, 63]. This questioned the possibility that APP-CTFs could be directly responsible for mitochondrial alterations observed in AD.

Our study is the first demonstration that, independently from Aβ, APP-CTFs accumulation either triggered by pharmacological blockade of its cleaving enzyme γ-secretase or after its overexpression leads to mitochondrial size alteration and cristae disorganization associated with enhanced mitochondrial ROS production, and mitophagy failure phenotype in cells. These alterations were similarly observed in γ-secretase inhibitor-treated young 3xTgAD mice and in adeno-associated-virus (AAV)-C99 injected mice. Importantly, we also reported mitophagy failure correlating with mitochondrial APP-CTFs accumulation in a cohort of human sporadic AD (SAD) brains. Together, our data demonstrate that APP-CTFs accumulation specifically drives mitochondrial dysfunctions and mitophagy failure, thus providing a cellular basis for counteracting setting or progression of AD pathology.

## Materials and methods

### Human brain samples

All procedures performed in studies involving human brains were in accordance with the ethical standards of the institutional and/or national research committee and with the 1964 Helsinki declaration and its later amendments or comparable ethical studies. Informed consent for tissue donation for research is obtained by the Brain Bank NeuroCEB and has been declared at the Ministry of Higher Education and Research (agreement AC-2013-1887) under their approval procedures. We studied control and SAD post-mortem human brains. Cases were anonymized, but information was provided regarding sex, age at death, and neuropathology (suppl. Table 1, online resource).

### Mice models, treatments, and analyses

3xTgAD (APPswe: KM670/671NL, TauP301L, and presenelin1 (PS1) M146V) and non-transgenic wild-type (WT) mice colonies were maintained from breeding pairs generously provided by Dr LaFerla [56]. As described previously, 2xTgAD expressing APPswe, TauP301L, and wild-type PS1 were obtained in our laboratory by crossing the 3xTgAD with WT mice [43]. Therefore, the same WT mice with the original hybrid background (129-C57BL/6) were used as control for both 2xTgAD and 3xTgAD mice. Each strain line is maintained by intercross breeding and backcrossed with the original strain every 10 generations. Males and females were housed with a 12:12 h light/ dark cycle and were given free access to food and water. All experimental procedures were in accordance with the European Communities Council Directive of 22 September 2010 (2010/63/EU) and approved by the French Ministry of Higher Education and Research (Project no: APAFIS#20495-201904231352370).

Five-month-old 3xTgAD and WT females were treated daily with vehicle (methylcellulose/polysorbate 80) or γ-secretase inhibitor (ELND006, Elan Pharmaceuticals, South San Francisco, CA [43]); (30 mg/kg) by oral gavage during 1 month. Animals were sacrificed 6 h after the last administration. We also used WT, 2xTgAD, and 3xTgAD males aged 17 months [11, 43], and new born males and females C57BL/6 mice injected with AAV-C99- or AAV-Free (empty virus) [45] and used at 2–3-month-old or 12-month-old. Mice brains were processed for SDS-PAGE, immunofluorescence or electron microscopy ultrastructure analyses.

For biochemical analyses, brains were isolated and stored in the RNA stabilization reagent (RNAlater, Qiagen) for 24 h and then dried and stored at − 80 °C until use. We used hippocampus of WT, 2xTgAD, and 3xTgD mice and total brains of AAV-Free and AAV-C99 injected mice.

For immunofluorescence and electron microscopy analyses, mice were anesthetized with a ketamine/xylazine (87 mg/ml and 13 mg/ml, respectively, 1 ml/kg) mixture and cardially perfused with PBS for 5 min and then with 10 ml PFA 4% solution (immunofluorescence) or with 10 ml of 2.5% glutaraldehyde in 0.1 M cacodylate buffer (electron microscopy analyses). The whole brains were isolated and further post-fixed overnight at 4 °C under agitation. For immunofluorescence, fixed brains were embedded in paraffin and coronal sections were cut with a sliding microtome (8 μm).

### Cell lines and treatments

Cell lines were cultured in Dulbecco’s modified Eagle’s medium supplemented with 10% fetal calf serum, penicillin (100 U/ml) and streptomycin (50 μg/ml), and incubated at 37 °C in a 5% CO_2_ atmosphere. Human SH-SY5Y cells stably expressing pcDNA3.1, full-length APP wild type (APPwt), or APPswe constructs were generated as already described [59], and maintained in the presence of 400 µg/ml geneticin (Gibco). Stable inducible SH-SY5Y cell line expressing C99 fragment was obtained by co-transfection of the Sleeping Beauty inducible vector (pSBtet SPC99) and the transposase SB100 as already described [42] and were maintained in the presence of 10 µg/ml puromycin (Sigma-Aldrich). C99 expression was induced by the addition of doxycycline (10 μg/ml) (Sigma-Aldrich) for 48 h. We also used human embryonic kidney (HEK) cells (CRL-1573, ATCC). HEK cells were transiently transfected with pcDNA3.1 empty vector or C99 construct [29] using jetPRIME (polyplus transfection) following the manufacturer’s instructions. Cells were analyzed 48 h post-transfection. Cells were treated with γ-secretase inhibitor ELND006 at 5 µM for 20 h, and β-secretase inhibitor at 30 µM for 20 h (Elan Pharmaceuticals, South San Francisco, CA [43]). For caspase-3 assays, cells were pretreated with staurosporine (STS) at 1 µM for 2 h (Sigma-Aldrich). For mitophagy analysis, cells were treated with deferiprone (DFP) (Sigma-Aldrich) at 1 mM for 20 h, or carbonyl cyanide m-chlorophenyl hydrazone (CCCP) (Millipore-Sigma; St Quentin Fallavier, France) at 1 µM for 6 h.

### Protein extraction and SDS-PAGE analysis

Total protein extracts were prepared using lysis buffer (50 mM Tris pH 8, 10% glycerol, 200 mM NaCl, 0.5% Nonidet p-40, and 0.1 mM EDTA) supplemented with protease inhibitors (Complete, Roche diagnostics). Mitochondrial fraction was isolated using isolation buffer (250 mM D-Mannitol, 5 mM HEPES pH 7.4, 0.5 mM EGTA, and 0.1% BSA) supplemented with protease inhibitor mixture. After chilling on ice for 20 min with frequent tapping, cells, mice brains, or dissected hippocampi were disrupted by 120 strokes of a glass Dounce homogenizer, and the homogenate was centrifuged at 1500 × *g* at 4 °C to remove unbroken cells and nuclei. Part of the supernatant was collected for total fraction, and the other part was centrifuged at 10,000 × *g* at 4 °C for 10 min to pellet mitochondrial fraction which was suspended in isolation buffer supplemented with protease inhibitors. Full-length APP, APP-CTFs, and Aβ were resolved on 16.5% Tris-Tricine SDS-PAGE then transferred onto nitrocellulose membranes. Membranes were boiled in PBS, saturated in TBS, 5% skimmed milk, and incubated overnight with specific antibodies (suppl. Table 2, online resource). All the other proteins were resolved by SDS-PAGE following standard procedures.

### Immunofluorescence and immunohistochemistry

Human and mice brain sections were deparaffined in xylen bath and rehydrated by successive 5 min baths of EtOH 100% (2 times), 90%, and then 70%. Antigens were unmasked in a 90% formic acid bath for 5 min for APP-Cter and 82E1 antibodies (Fig. 10g), or for 30 min in a pressure cooker with pH6 citric acid solution (Vector Laboratories) for APP-Cter and TIMM23 antibodies co-staining (Fig. 8c). Non-specific binding was blocked for 1 h in 5% BSA, 0.05% Triton in PBS solution. Sections were incubated at 4 °C overnight with primary antibodies (suppl. Table 2, online resource). After washes, sections were incubated with secondary antibodies [HRP-conjugated (1:1000; Jackson ImmunoResearch) or fluorescent Alexa Fluor antibodies, and Alexa 488- and Alexa 594-conjugated (Invitrogen; 1:1000)] at room temperature during 1 h. Nuclei were revealed with DAPI (Roche; 1:20,000). Immunofluorescence was visualized with SP5 confocal microscopes. Slides with HRP-conjugated antibodies were incubated with DAB-impact (Vector), rinsed, and counterstained with cresyl violet, and analyzed using an optical light microscope (DMD108; Leica).

Cells were plated directly on glass coverslip (pre-washed with 70% ethanol) and cultured as described previously. Transfected and/or treated cells were washed with PBS and then fixed with 4% PFA 20 min. Cells were permeabilized with 0.5% Triton-PBS and finally incubated with a 3% BSA blocking solution for 1 h. Coverslips were covered with a solution of 0.3% BSA with diluted primary antibodies overnight (suppl. Table 2, online resource). Fluorescent secondary antibodies were added after PBS wash for at least 1 h and nuclei were stained with Dapi (1/10,000). Coverslips were mounted on glass slides with Vectamount medium (Vector). Images were acquired with SP5 confocal microscope and processed using ImageJ software.

### Measurements of mitochondrial superoxide and of mitochondrial potential

MitoSOX Red is a fluorogenic dye for highly selective detection of superoxide in the mitochondria of living cells [70]. Cells were incubated in 5 µM MitoSOX red mitochondrial superoxide indicator (Invitogen) in DMEM for 30 min, at 37 °C. Cells were harvested and rinsed twice with ice-cold HBSS complemented with 1 mM CaCl_2_  and 0.5 mM MgCl_2_. Tetramethyl rhodamine methyl ester (TMRM) is a cell-permanent dye that accumulates in active mitochondria with intact membrane potentials. Loss of mitochondrial membrane potential triggers reduced TMRM accumulation. Cells were harvested, rinsed with PBS, and incubated in TMRM (prepared in DMEM) (2 nM) for 30 min at 37 °C. TMRM and MitoSox fluorescence median intensities were then analyzed in the Novocyte Flow Cytometer (ACEA bioscience, Inc) excitation/emission 510 nm/580 nm. TMRM fluorescence signal was also acquired on a Leica SP5 confocal microscope with 63 × objective, after loading cells with TMRM (2 nM) in KRB/1 mM CaCl_2_ for 30 min at 37 °C. To demonstrate specific TMRM binding, measurements were corrected for residual TMRM fluorescence after full mitochondrial membrane collapse with the mitochondrial uncoupler FCCP (trifluoromethoxy carbonylcyanide phenylhydrazone) [22].

### Transmission electron microscopy

For ultrastructure analysis, cells were fixed in 1.6% glutaraldehyde in 0.1 M phosphate buffer (pH 7.4), rinsed with cacodylate buffer 0.1 M, and then post-fixed in osmium tetroxide (1% in cacodylate buffer) reduced with potassium ferrycyanide (1%) for 1 h. Fixed mice brains were sliced (200 µm) on a vibratome. Two mm^3^ cubes from the cortical regions or subiculum were microdissected under binoculars and post-fixed in osmium tetroxide (1% in cacodylate buffer 0.1 M). Cells and tissue were dehydrated with several incubations in increasing concentrations of ethanol or acetone, respectively, and embedded in epoxy resin (EPON), and 70 nm ultrathin sections were contrasted with uranyl acetate and lead citrate and observed with a Transmission Electron Microscope (JEOL JEM 1400) operating at 100 kV and equipped with a Olympus SIS MORADA camera. We used ImageJ software to analyze mitochondria ultrastructure and to measure mitochondria perimeter, area and number.

### Respiratory chain complexes activity

Enzymatic spectrophotometric measurements of the OXPHOS respiratory chain complexes and citrate synthase were performed at 37 °C on cells according to standard procedures [73]. The Krebs cycle enzyme citrate synthase was used as control for similar mitochondria content between samples [73].

### Caspase 3 activity

Cells were cultured in 6-well plates and then incubated for 24 h at 37 °C in the presence or absence of STS. In some cases, cells were either pre-incubated overnight with γ-secretase inhibitor ELND006. Caspase 3 activity was then measured as described [18]. Fluorometry was recorded at 390 and 460 nm for excitation and emission wavelengths, respectively, by means of a plate reader (Varioskan, ThermoFisher scientific). Caspase3-like activity was calculated from the linear part of fluorimetry recording and expressed in units/h/mg of proteins. One unit corresponds to 4 nmol of amidomethylcoumarin released.

### Mitophagy analysis

Cells were transfected with Cox8-EGFP-mCherry (a gift from David Chan; Addgene plasmid # 78520 [71]), LC3-GFP probe or co-transfected with Lamp1-GFP (a gift from Falcon-Perez; Addgene plasmid # 34831 [26]), and Mit-RFP probes using lipofectamine. Cells were treated 24 h after transfection and analyzed 24 h later. The Cox8-EGFP-mCherry mitophagy reporter is based on differences in pKa of green fluorescent protein (EGFP), and mCherry expressed in tandem with the mitochondrial localization signal of Cox8. Under normal conditions, mitochondria fluoresce red and green (yellow colocalization signal). In mitophagy, mitochondria are delivered to lysosomes where the low pH quenches the EGFP signal (scheme in Fig. 5h). The result is that a portion of mitochondria fluoresce red only. Data are presented as the percentage of cells undergoing mitophagy. A threshold of a single or more red-alone puncta per cell was applied to all cells expressing Cox8-EGFP-mCherry probe. LC3-GFP transfected cells were subjected to immunostaining with HSP60 antibody. Data are presented as the percentage of cells harboring diffuse versus dotted LC3-GFP signal. The colocalization of Lamp1-GFP with Mit-RFP was determined using ImageJ plug-in JACoP (Just Another Colocalization Plug-in [9]. Data represent Mander’s coefficient of Mit-RFP with LAMP1-GFP signal. Cells were observed with Leica SP5 or Zeiss LSM 780 with 63X Objective.

### Statistical analyses

Data were expressed as means ± SEM. Sample size for each experiment is indicated in the figure captions. We studied control (*n* = 9) and SAD (*n* = 15) human brains by SDS-PAGE and SAD human brains (*n* = 6) by immunohistochemistry. Cells analyses were obtained in at least three independent experiments in duplicates or triplicates (unless were indicated), while the quantification of imaging experiments was performed on different (> 5) fields of view. SDS-PAGE analyses using mice brains/hippocampi were performed in 4–7 different mice, while immunofluorescence and electron microscopy analyses were performed in 2–3 different mice. Data were analyzed with GraphPad Prism version 8 for Windows (GraphPad Software, La Jolla, CA, USA; https://www.graphpad.com). Data were first analyzed for normal distribution. We used the Mann–Whitney test when the two groups of variables have not passed the normality test. Groups of more than two variables that have passed normality test were analyzed by one-way ANOVA with Dunnett’s or Tukey’s multiple comparisons post-test. Kruskal–Wallis test and Dunn’s multiple comparisons post-test was used when groups of variables have not passed the normality test. Correlations were analyzed with Spearman’s correlation coefficient. Significant differences are: *P < 0.05, **P < 0.01, ***P < 0.001, ****P < 0.0001 and ns: non-significant.

## Results

### Mitochondrial structure and function are altered in a cellular model of familial AD

**Fig. 1:**
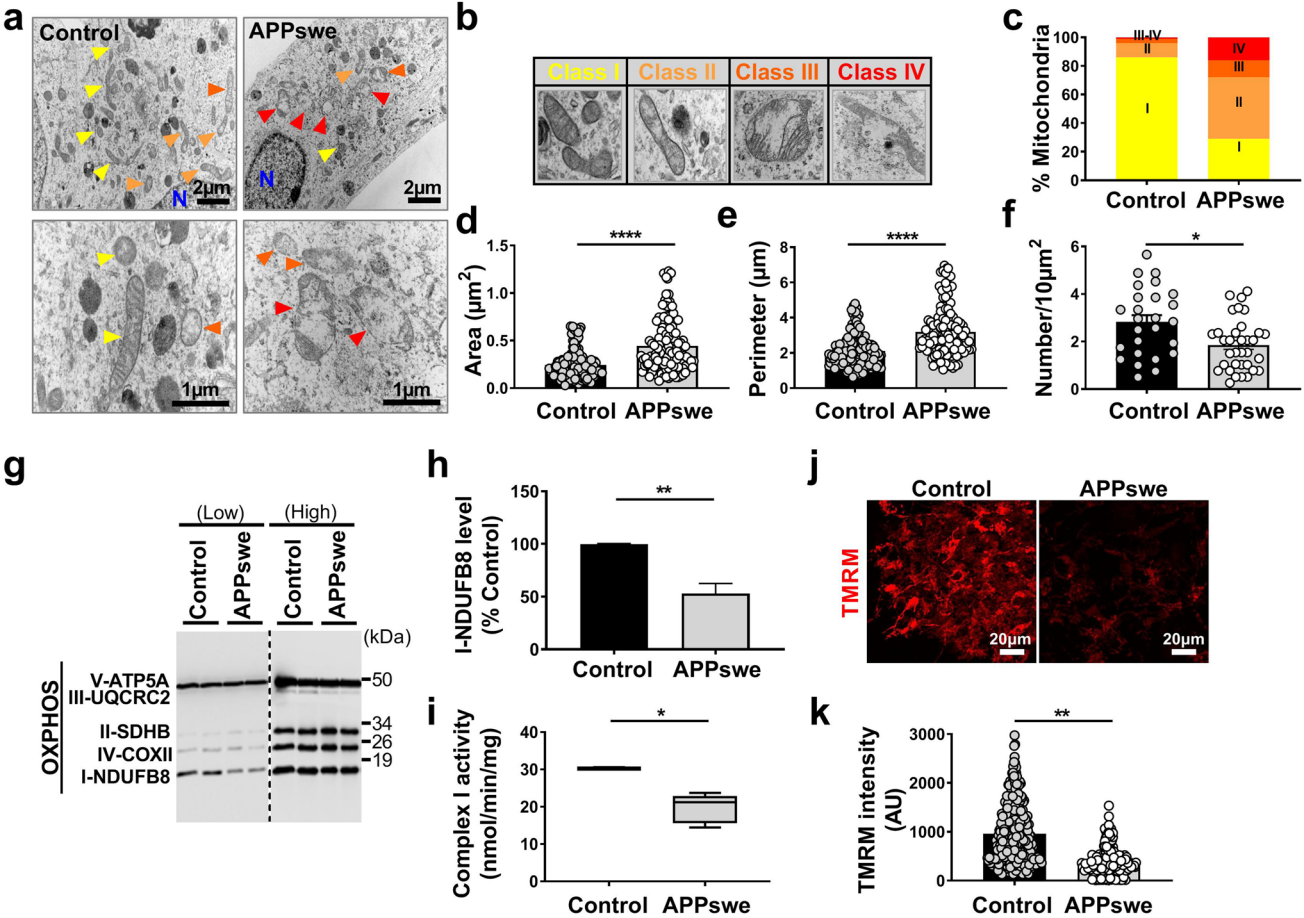
SH-SY5Y cells expressing APPswe display altered mitochondrial structure and function. **a** Electron microscopy ultrastructure of SH-SY5Y cells stably transfected with pcDNA3.1 empty vector (control) or with APPswe cDNA (APPswe). Scale bars correspond to 2 or 1 µm. *N* nucleus. Colored arrowheads indicate mitochondria. Representative images of mitochondria classes I, II, III, and IV (**b**), and their quantitative distribution (**c**) in control and APPswe cells. Quantitative graphs of the means ± SEM of mitochondria area (µm^2^) (**d**), perimeter (µm) (**e**), and number/10 µm^2^ (**f**). **a**–**f** Data were obtained in two independent experiments performed in duplicates. The quantification was done in at least 20 different fields (> 150 mitochondria). **g** SDS-PAGE of mitochondrial OXPHOS subunits at low and high exposures revealed in total cell extracts revealed using OXPHOS antibodies mix. **h** Complex I-NDUFB8 subunit expression presented as means ± SEM (*n* = 4) of control (taken as 100%). **i** Spectrophotometric analysis of the respiratory chain complex I activity expressed as absolute values in nanomols of substrate/min/mg of proteins and presented as means ± SEM (*n* = 3). Confocal images of TMRM fluorescence (**j**) (scale bars represent 20 µm), and quantification of TMRM fluorescence intensity (*AU* arbitrary units) (**k**) (*n* = 3, > 150 cells). *P < 0.05, **P < 0.01, and ****P < 0.0001 versus control using Mann–Whitney test

We analyzed mitochondrial structure and function in the neuroblastoma cells expressing the Swedish familial double mutations (APPKM670/671NL: SH-SY5Y APPswe). These cells exacerbate the production of APP-CTFs (C99 and C83 derived from the cleavage of APP by β and α secretases, respectively), and amyloid beta (Aβ) peptides (produced through the subsequent cleavage of C99 by γ-secretase) [59]. We examined mitochondria morphology and size by transmission electron microscopy. We first observed that APPswe-expressing cells harbor larger size mitochondria with altered cristae organization than control cells (colored arrowheads in Fig. 1a). We analyzed in depth mitochondrial alterations and classified mitochondria morphology in four categories (class I: fairly dark mitochondria, with uniform matrix filled with dense packed regular distributed cristae; class II: mitochondria with disrupted cristae and loss of matrix density; class III: empty mitochondria with disorganized cristae, or cristae on the periphery; and class IV: swollen mitochondria with disrupted membrane) (Fig. 1b). Quantification of mitochondria subclasses was then recorded revealing that while control cells exhibited 86% of mitochondria class I, 10% class II, 3% class III, and 1% class IV (Fig. 1c and suppl. Table 3, online resource), APPswe cells displayed a drastic reduction of “healthy” mitochondria class I (29%), and a concomitant enhancement of mitochondria class II (43%), III (12%), and IV (16%) (Fig. 1c and suppl. Table 3, online resource). We then showed that APPswe cells also displayed larger mitochondria (increased area and perimeter) (Fig. 1d, e and suppl. Table 3, online resource), with a significant decrease of mitochondrial number (Fig. 1f and suppl. Table 3, online resource) as compared to control cells.

Mitochondria are dynamic organelles requiring appropriate finely tuned equilibrium between fission and fusion. To corroborate structural alterations observed in APPswe cells, we measured the levels of mitochondrial fission protein (DNM1L/DRP; dynamin 1-like) and fusion proteins (MFN1; mitofusin 1, and MFN2; mitofusin 2) at the mRNA and protein levels (Suppl. Figure 1a-e, online resource). While we did not reveal a modulation of DRP1, MFN1, and MFN2 mRNAs levels between APPswe cells and their controls (Suppl. Figure 1a, online resource), the protein levels of DRP1, MFN1, and MFN2 were significantly lower in APPswe cells in both total and mitochondria-enriched protein extracts (Suppl. Figure 1b-e, online resource). These data show molecular perturbations of mitochondrial fission/fusion equilibrium supporting mitochondria morphological phenotypes taking place in APPswe cells.

Mitochondria structure alterations observed in APPswe cells are accompanied by several mitochondrial dysfunctions as demonstrated by a reduction of mitochondrial complex I NDUFB8 subunit expression (Fig. 1g, h), while the levels of mitochondrial complexes subunits II, III, IV, and V remained unchanged (Fig. 1g and suppl. Figure 1f, online resource). This was corroborated by a specific reduction of mitochondrial complex I activity (Fig. 1i), but not of complexes II, III, IV, and V (Suppl. Figure 1g-j, online resource) in APPswe cells. The Krebs cycle citrate synthase enzyme remained also unchanged between APPswe cells and their controls (Suppl. Figure 1k, online resource). In agreement with a dysfunctional respiratory chain complex I, APPswe cells showed a reduction of mitochondrial membrane potential, revealed by reduced TMRM fluorescence intensity (Fig. 1j, k).

### APP-CTFs accumulation triggers mitochondrial structure alterations in cells independently of Aβ

**Fig. 2:**
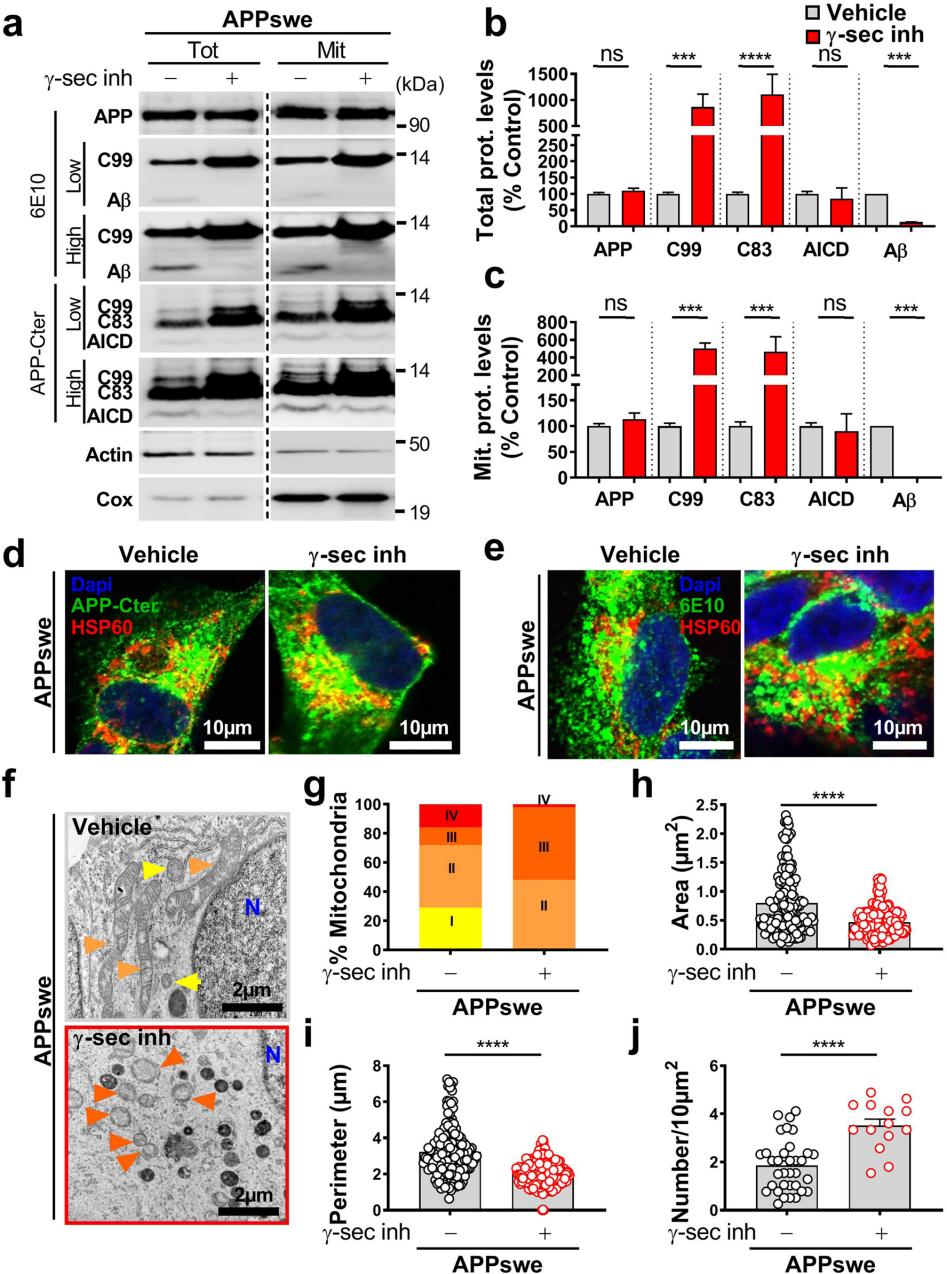
APP-CTFs accumulate in mitochondria and trigger mitochondria structure alteration in cells.** a** SDS-PAGE (low and high exposures) of APP and its indicated metabolites in vehicle (−)- or γ-secretase inhibitor- (γ-sec inh) ( +) (5 µM, 20 h) treated APPswe cells revealed in total cell extracts (Tot), and mitochondrial-enriched fraction (Mit). Full-length APP (APP), C99, and Aβ detected with 6E10 antibody, and C99, C83, and AICD detected with APP-Cter antibody. Actin and Cox IV antibodies were used as loading controls for total and mitochondria-enriched fractions, respectively. Quantitative graphs of indicated proteins in total extracts (**b**) and mitochondrial fraction (**c**) expressed as means ± SEM (*n* = 5) versus controls (taken as 100%). **d, e** Immunostaining of SH-SY5Y APPswe cells treated as in (**a**) with APP-Cter or 6E10 antibodies (green) in combination with anti-HSP60 (red) used to stain mitochondria. Nuclei were labeled with DAPI. Images show merge of green and red signals reflecting the colocalization in yellow. Scale bars represent 10 µm. **f** Electron microscopy ultrastructure of SH-SY5Y APPswe-treated as in (**a**). Scale bars correspond to 2 µm. *N* nucleus. Colored arrowheads indicate mitochondria classes as shown in Fig. 1b. Quantitative graphs of mitochondria classes (**g**), and means ± SEM of mitochondria area (µm^2^) (**h**), perimeter (µm) (**i**), and number/10 µm^2^ (**j**). **f**–**j** The quantification was obtained in two independent experiments performed in duplicates in at least 20 different fields (> 150 mitochondria). **b**, **c** ***P < 0.001, ****P < 0.0001, and *ns* non-significant using Kruskal–Wallis test and Dunn’s multiple comparison post-test. **h**–**j** ****P < 0.0001 versus vehicle (−) using Mann–Whitney test

Several studies reported that mitochondrial structure and function alterations are linked to Aβ [15, 34, 46]. We aimed hereafter at investigating the specific contribution of APP-CTFs versus Aβ to mitochondrial structure and function alterations reported in our APPswe cellular model. Thus, we pharmacologically targeted γ-secretase, the inhibition of which blocks Aβ peptide formation and enhances CTFs’ recovery (as we already reported [13, 45]). Immunological analyses with 6E10 antibody (revealing full-length APP, C99, and Aβ) or APP-Cter antibody (revealing C99, and C83, and APP intracellular domain (AICD) (Fig. 2a) confirmed that γ-secretase inhibition enhanced the accumulation of APP-CTFs (C99 and C83) and blocked Aβ peptide production in total extracts (Fig. 2a, b) as well as in mitochondria-enriched fraction (Fig. 2a, c) without affecting the level of full-length APP (Fig. 2a–c). Unexpectedly, γ-secretase inhibition did not significantly modify AICD levels in both total homogenates and mitochondrial-enriched fraction (Fig. 2a–c). We further demonstrated by immunofluorescence analyses, using the two sets of antibodies (6E10 and APP-Cter), that APP and APP-CTFs colocalize with the mitochondrial protein HSP60 in untreated and in γ-secretase inhibitor-treated APPswe cells (Fig. 2d, e). These biochemical and imaging analyses firmly demonstrate that APP-CTFs are present in mitochondria compartment, thus questioning their contribution to mitochondrial structure alterations observed in this AD cellular model.

We took advantage of this pharmacological approach and analyzed mitochondria structure using electron microscopy, and revealed that γ-secretase inhibitor-treated APPswe cells display spherical and fragmented mitochondria (colored arrowheads in representative images in Fig. 2f). The quantification of mitochondria subclasses showed that γ-secretase inhibitor-treated APPswe cells harbor a loss of mitochondria class I (1%) and swollen mitochondria class IV (2%) with a drastic shift of mitochondrial shape towards mitochondrial classes II and III (47%, and 50%, respectively) (Fig. 2g and suppl. Table 3, online resource). This mitochondrial morphology shift was accompanied by a significant reduction in mitochondria size (area and perimeter) (Fig. 2h, i and suppl. Table 3, online resource), and an increase of mitochondria number in γ-secretase inhibitor-treated APPswe cells (Fig. 2j and suppl. Table 3, online resource). We further strengthened these observations by analyzing mitochondrial three-dimensional (3D) structure in living cells using mitotracker dye and confocal imaging (suppl. Figure 2a, online resource). Thus, γ-secretase inhibition increases mitochondrial number (Suppl. Figure 2a, b, online resource), and reduces mitochondrial network 3D volume (Suppl. Figure 2a, c, online resource). As observed in APPswe untreated cells (Suppl. Figure 1a, online resource), mitochondrial size alteration observed upon γ-secretase inhibition is not corroborated by a modulation of DRP1 and MFN2 mRNA levels (Suppl. Figure 2d, online resource). Nevertheless, we reported a significant increase of MFN2 protein level in APPswe cells treated with γ-secretase inhibitor, while DRP1 protein level remained unchanged (Suppl. Figure 2e, online resource).

We further investigated the contribution of APP-CTFs versus Aβ to mitochondria structure alterations by analyzing APPswe cells treated with β-secretase inhibitor, blocking the production of Aβ, reducing APP-βCTF (C99) level, and enhancing the level of APP-αCTF (C83) (Suppl. Figure 3a-c, online resource). As for γ-secretase inhibitor, we also did not notice any significant change of AICD level upon β-secretase inhibition (Suppl. Figure 3a-c, online resource). Importantly, we revealed by electron microscopy analyses that β-secretase inhibition triggers a recovering of mitochondria class I morphology (76%) and a reduction of mitochondria classes II, III, and IV (13%, 7%, and 4%, respectively, suppl. Figure 3d, e, online resource, and suppl. Table 3, online resource). Thus, the comparative analyses of the impact of β- versus γ-secretase inhibitors on mitochondria structure demonstrate that both Aβ and APP-CTFs participate to mitochondrial structure alterations in APPswe-expressing cells and that γ-secretase-mediated APP-CTFs accumulation independently from Aβ exacerbate mitochondrial morphological alterations specifically characterized by cristae disorganization (mitochondria classes II and III) and changes in mitochondria size and number.

We also investigated the impact of the accumulation of endogenous APP-CTFs on mitochondrial structure upon γ-secretase inhibition in mock (pcDNA3.1)-transfected SH-SY5Y cells. We revealed low amount of endogenous APP-CTFs in both total and mitochondrial fraction of mock-transfected SH-SY5Y cells (as compared to APPswe cells) correlating with a low-level expression of full-length APP (Suppl. Figure 4a, online resource). We also noticed the accumulation of endogenous APP-CTFs in control cells upon γ-secretase inhibitor treatment both in total and mitochondria-enriched fraction (Suppl. Figure 4b, online resource). Immunofluorescence analyses support this finding and show enhanced signal detected with APP-Cter antibody colocalizing with mitochondrial protein HSP60 in γ-secretase inhibitor-treated-control cells (Suppl. Figure 4c, online resource). We then revealed that endogenous APP-CTFs accumulation led to mitochondria structure alteration (66% class II, and 30% class III) (Suppl. Figure 4d, e, online resource, and suppl. Table 3, online resource), reduced size (Suppl. Figure 4d, f, g, online resource, and suppl. Table 3, online resource), and increased number (Suppl. Figure 4h, online resource, and suppl. Table 3, online resource). Thus, γ-secretase inhibition that triggers accumulation of endogenous or overexpressed APP-CTFs leads to similar structural mitochondria alterations in cells (Fig. 2f–j, suppl. Figure 4d-h, online resource, and suppl. Table 3, online resource).

### Mitochondrial function and apoptosis are differently impacted by Aβ and APP-CTFs

**Fig. 3:**
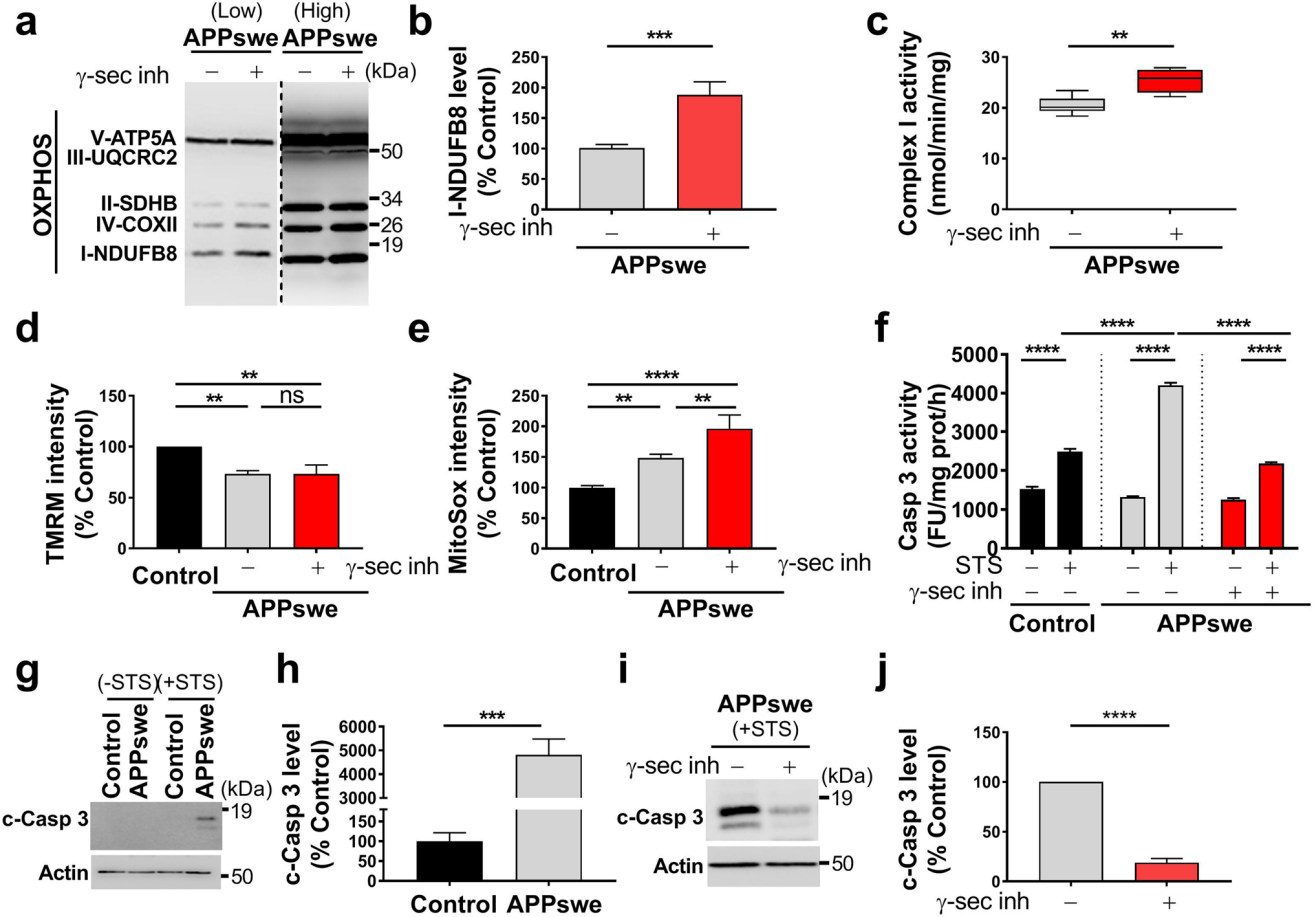
APP-CTFs accumulation triggers mitochondria dysfunction in cells. **a** SDS-PAGE (low and high exposures) of mitochondrial OXPHOS subunits observed in total cell extracts of SH-SY5Y APPswe cells treated with vehicle (−) or γ-secretase inhibitor (γ-sec inh) ( +) (5 µM, 20 h). **b** Complex I-NDUFB8 subunit expression presented as means ± SEM (*n* = 5) of control cells (taken as 100%). **c** Spectrophotometric analysis of the respiratory chain complex I activity expressed as absolute values in nanomols of substrate/min/mg of proteins and presented as means ± SEM (*n* = 4). **d, e** TMRM median intensity (**d**) and MitoSox median intensity (**e**) obtained by FACS analyses in control and APPswe cells treated as in (**a**). Data are expressed as means ± SEM (*n* = 3–5) of control (taken as 100%). **f** Caspase 3-like activity under basal and staurosporine (STS) stimulation (1 µM, 2 h) in the same experimental conditions as in (**d**) and expressed as fluorescence intensity (fluorescence units (FU)/mg protein/h) ± SEM (*n* = 6–7). **g-j** SDS-PAGE (**g**, **i**) and quantitative graphs (**h**, **j**) of cleaved caspase-3 (c-Casp 3) band in the same experimental conditions as in (**f**). Actin was used as loading control. **h, j** c-Casp levels are expressed as means ± SEM (n = 4–6) of respective control conditions (taken as 100%). **b, h, j** ***P < 0.001, ****P < 0.0001 versus control or vehicle-treated APPswe cells using *t* test. **c** **P < 0.01 versus vehicle-treated APPswe cells using Mann–Whitney test. **d, e, f** **P < 0.01 **** P < 0.0001, and ns: non-significant using one-way ANOVA and Tukey’s multiple comparison post-test

We investigated the consequences of mitochondrial APP-CTFs accumulation on mitochondrial function. We first showed that both γ-secretase and β-secretase inhibition enhanced the expression level of NDUFB8 complex I subunit (Fig. 3a, b and suppl. Figure 3f, online resource), and accordingly the activity of respiratory complex I (Fig. 3c and suppl. Figure 3g, online resource), without altering the expression levels (Suppl. Figure 5a, online resource) or activities (Suppl. Figure 5b-e, online resource) of mitochondrial complexes II, III, IV, and V. Citrate synthase enzyme remained also unchanged upon γ-secretase inhibition (Suppl. Figure 5f, online resource). Similarly, we did not notice any change in mitochondrial complexes II, III, IV, and V expression and activities upon β-secretase inhibition (data not shown). We then reported that reduced mitochondrial potential in untreated APPswe cells (compared to their controls) was unaffected by γ-secretase inhibitor (Fig. 3d and representative histograms in suppl. Figure 5g, online resource), but was restored by β-secretase inhibitor (suppl. Figure 3h, online resource). However, we observed enhanced mitochondrial ROS accumulation in APPswe versus control cells that was further exacerbated upon γ-secretase inhibition (Fig. 3e and suppl. Figure 5h, online resource), and unchanged upon β-secretase inhibition (data not shown).

We then quantified caspase*-*3 activation known as an executioner caspase in mitochondrial-dependent apoptosis and used fluorometric and immunoreactivity approaches [18]. We first showed higher sensitivity to a sub-apoptotic dose of staurosporine in APPswe cells as compared to control cells as illustrated by enhanced caspase-3 activity and cleaved caspase-3 (c-Casp 3: active form) (Fig. 3f–h). Although γ-secretase inhibitor did not impact caspase-3 activity in basal conditions (Fig. 3f), it triggers a significant reduction of STS-induced caspase-3 activity (Fig. 3f), and cleavage (Fig. 3i, j) in APPswe cells. We also confirmed the reduction of caspase-3 activity with two different γ-secretase inhibitors DFK, and DAPT in STS-treated APPswe cells (Suppl. Figure 5i, online resource). Similarly, we also observed a reduction of STS-induced caspase-3 activity upon treatment with β-secretase inhibitor (Suppl. Figure 3i, online resource).

We ascertain the specificity of γ-secretase inhibitor towards APP substrate using SH-SY5Y control cells and mouse embryonic fibroblasts (MEF) isolated from control mice (MEF-WT) or from mice genetically invalidated for both APP and its additional family member APLP2 gene (MEF-APPKO) (Suppl. Figure 6, online resource). We showed that γ-secretase inhibitor: (1) did not significantly change mitochondrial potential in SH-SY5Y control cells (Suppl. Figure 6a, online resource), and in MEF-WT and MEF-APPKO cells (Suppl. Figure 6b, online resource); (2) did not change STS-induced caspase-3 activity in MEF control and APPKO fibroblasts (Suppl. Figure 6e, online resource). Importantly, we reported increased mitochondrial ROS in SH-SY5Y control cells (Suppl. Figure 6c, online resource) and in MEF-WT cells but not in MEF-APPKO cells (Suppl. Figure 6d, online resource) treated with γ-secretase inhibitor. Thus, γ-secretase inhibitor-induced mitochondrial deleterious phenotype appears to be APP-dependent.

Overall, these results revealed the contribution of both Aβ and APP-CTFs to mitochondrial dysfunctions. The comparative analyses of the impact of γ-secretase versus β-secretase inhibitors in APPswe cells emphasized the role of Aβ in mitochondrial respiratory chain complex I dysfunction, mitochondrial membrane potential loss, and caspase-3 activation and that of APP-CTFs accumulation in mitochondrial ROS elevation.

**Fig. 4:**
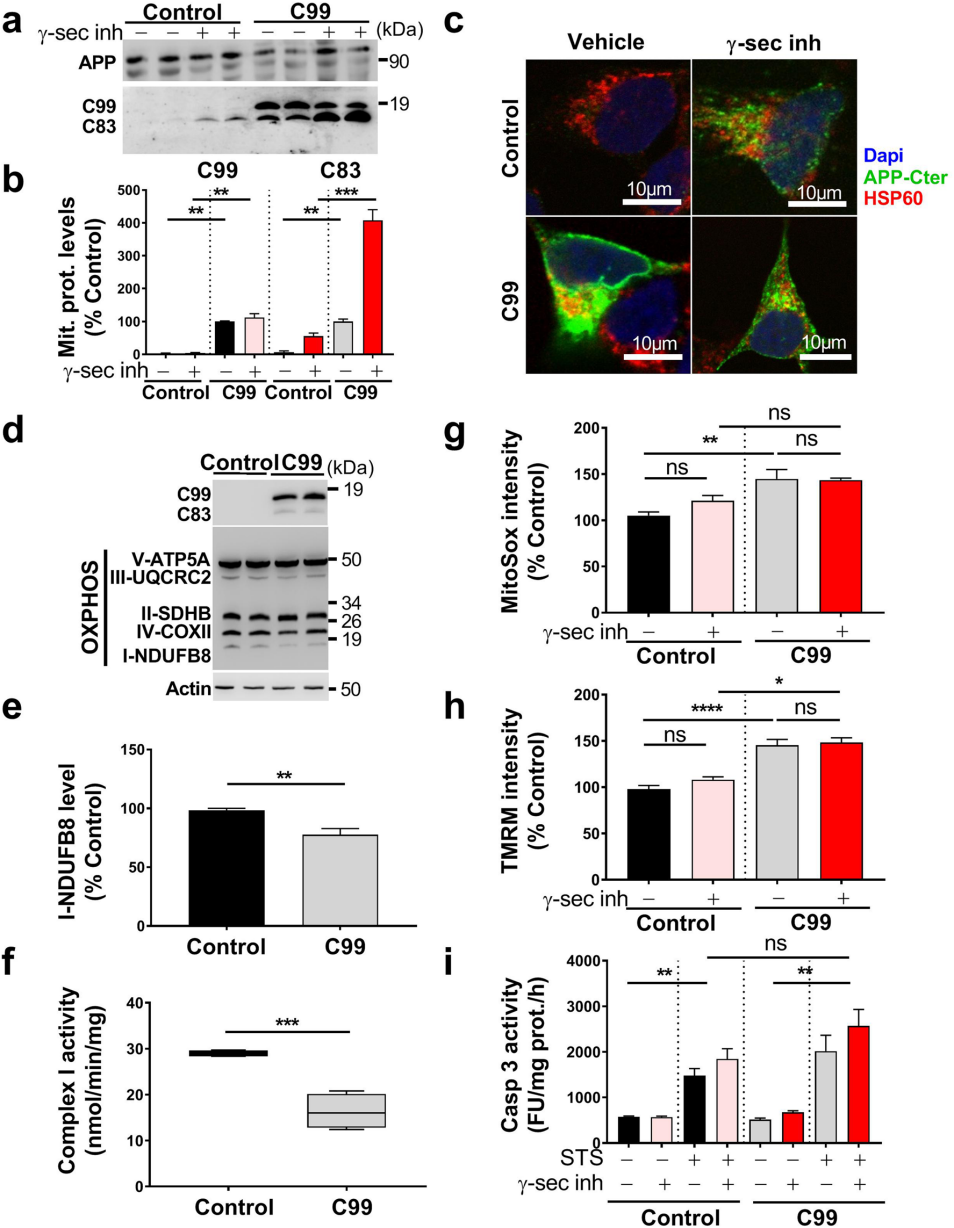
Overexpressed C99 localizes to mitochondria and leads to mitochondrial function alterations. **a** SDS-PAGE of doxycycline-induced (10 µg/ml, 24 h) or control pSBtet SPC99-transfected SH-SY5Y cells treated with vehicle (−) or with γ-secretase inhibitor ( +) (γ-sec inh; 5 µM, 20 h). Full-length APP (APP), C99, and C83 were detected in mitochondrial-enriched fraction using APP-Cter antibody. **b** Quantification of C99 and C83 levels in mitochondrial fraction presented as means ± SEM versus C99 cells treated with vehicle (−) (taken as 100%) and obtained in four independent experiments. **c** Immunostaining of SH-SY5Y C99 and control cells treated as in (**a**) with APP-Cter (green) and HSP60 (red) antibodies. Images show merge of green and red signals reflecting the colocalization in yellow. Nuclei were labeled with DAPI. Scale bars represent 10 µm. **d** SDS-PAGE of C99 and C83 and of mitochondrial OXPHOS subunits obtained in total extracts of HEK cells transiently transfected with either pcDNA3.1 (control) or C99 cDNA. Actin was used as loading control. **e** Complex I-NDUFB8 subunit expression presented as means ± SEM (*n* = 5) versus control (taken as 100%). **f** Spectrophotometric analysis of complex I activity in HEK cells transfected as in (**d**) and expressed as means ± SEM (*n* = 4) of absolute values in nanomols of substrate/min/mg of proteins. Graphs representing MitoSox (**g**) or TMRM (**h**) median intensities obtained by FACS analyses in control and C99 cells treated as in (**a**). Median fluorescence intensities are expressed as means ± SEM (*n* = 3 in triplicates) versus control treated with vehicle (−) (taken as 100%). **i** Caspase 3-like activity was assessed under basal condition and STS stimulation (1 µM, 2 h) in cells treated as in (a). The graph represents means of fluorescence intensity (Fluorescence units (FU)/mg protein/h) ± SEM (*n* = 2 in triplicates). **b** **P < 0.01, ***P < 0.001 using Kruskal–Wallis test and Dunn’s multiple comparison post-test. **e, f** **P < 0.01, ***P < 0.001 versus control using *t* test. **g**–**i** *P < 0.05, **P < 0.01, ****P < 0.0001 and ns: non-significant using one-way ANOVA and Tukey’s multiple comparison post-test

To further explore the contribution of APP-derived CTFs to mitochondrial dysfunctions, we used the neuroblastoma stable and inducible cell line expressing C99 fragment only [45]. First, as we previously described [29], we observed that C99 undergoes a γ-secretase-mediated cleavage giving rise to a high C83 expression (Fig. 4a, b). We also showed enhanced C99 and C83 fragments in mitochondrial-enriched fraction prepared from C99-expressing cells as compared to mock-transfected cells (control) (Fig. 4a, b). As observed in APPswe cells (Fig. 2a–c), the treatment with γ-secretase inhibitor enhanced C83 level in both C99 and control cells (Fig. 4a, b). We further demonstrated that both endogenous and overexpressed APP-CTFs colocalize with mitochondrial HSP60 protein as illustrated in C99 cells untreated and in control and C99 cells treated with γ-secretase inhibitor (Fig. 4c). We next examined whether C99 expression triggers mitochondrial dysfunction. Indeed, like APPswe cells, C99-expressing cells harbor a reduction of the expression of NDUFB8 (Fig. 4d, e) and of the activity of respiratory chain complex I (Fig. 4f), without altering the mitochondrial complexes II, III, IV, and V subunit expression and their activities (Suppl. Figure 7a–e, online resource), and citrate synthase activity (Suppl. Figure 7f, online resource). C99 overexpression triggers an increase of mitochondrial ROS production (Fig. 4g), and intriguingly enhanced mitochondrial membrane potential (Fig. 4h), both remained elevated upon γ-secretase inhibitor treatment. We also revealed that C99-expressing cells harbor a slight but not-significant increase in staurosporine-induced apoptosis that remained not modulated by γ-secretase inhibitor (Fig. 4i). All over, these data demonstrate the colocalization of overexpressed C99 with mitochondria triggering mitochondrial dysfunctions, and that APP-CTFs accumulation per se did not lead to apoptotic cell death.

### APP-CTFs accumulation leads to basal mitophagy failure in cells independently of Aβ

**Fig. 5:**
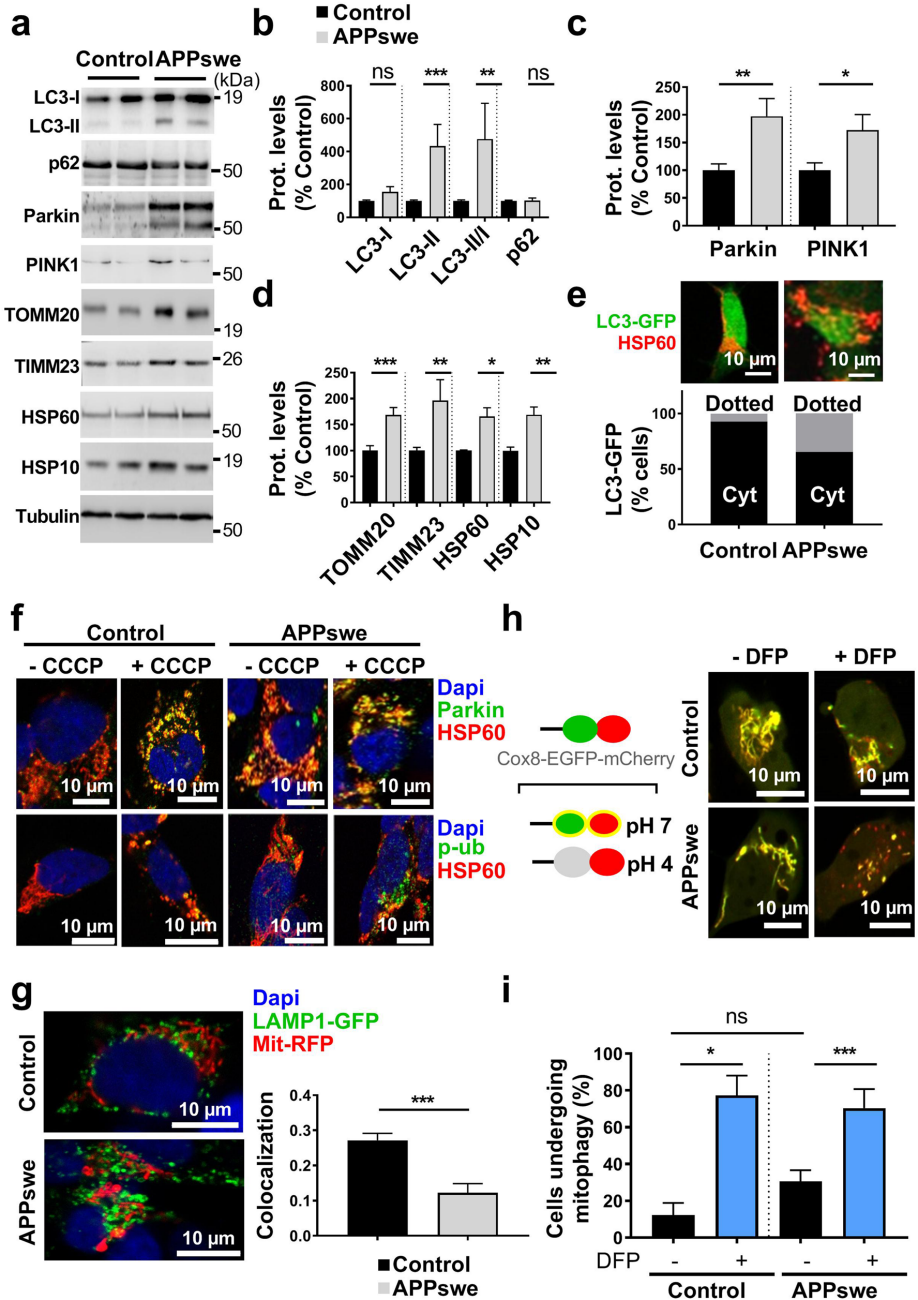
SH-SY5Y cells expressing APPswe display mitophagy failure phenotype. **a** SDS-PAGE of LC3-I and LC3-II, SQSTM1/p62 (p62), Parkin, PINK1, TOMM20, TIMM23, HSP60, and HSP10 in mitochondria-enriched fraction of SH-SY5Y cells stably transfected with pcDNA3.1 empty vector (control) and APPswe construct. Tubulin was used as loading control. **b**–**d** Quantitative graphs of indicated proteins expressed as means ± SEM (*n* = 3–4 in duplicates) versus control (taken as 100%). **e** Representative images of control and APPswe cells transiently transfected with LC3-GFP reporter (Green) stained with anti-HSP60 (Red). Scale bar represents 10 µm. The graph represents percentage of cells (*n* = 20–30) harboring cytosolic diffuse LC3-GFP signal (Cyt), or aggregated LC3-GFP signal (dotted yellow signal reflecting the colocalization of LC3-GFP and HSP60). **f** Immunostaining of control and APPswe cells treated with vehicle (− CCCP) or with CCCP ( +) (1 µM, 6 h) and stained with anti-Parkin or anti-phospho-poly-ubiquitin (p-S65-Ub: p-Ub) (green) and anti-HSP60 (red). **g** Representative images of control and APPswe cells transfected with LAMP1-GFP probe (green) and Mit-RFP probe (red). The graph shows the colocalization (Mander’s coefficient) of Mit-RFP with LAMP1-GFP, means ± SEM of *n* = 14 cells. **f**, **g** Nuclei were labeled with DAPI. Images show merge of green and red signals reflecting the colocalization in yellow. Scale bars represent 10 µm. **h** Scheme of Cox8-EGFP-mCherry mitophagy reporter construct (left). Representative images of vehicle- (−) or deferiprone (DFP)- ( +) (1 mM, 20 h) treated control and APPswe cells transiently transfected with Cox8-EGFP-mCherry probe (Right). **i** Graph represents percentage of cells (Means ± SEM of *n* = 55–130 cells) undergoing mitophagy (cells with fragmented and red mitochondria reflecting the mCherry signal only due to EGFP signal quenching at pH4). **b–d**, **g** *P < 0.05, **P < 0.01, ***P < 0.001, and ns: non-significant versus control using Mann–Whitney test. **i** *P < 0.05, ***P < 0.001, and ns: non-significant using Kruskal–Wallis test and Dunn’s multiple comparison post-test

Mitochondrial fragmentation and enhanced ROS production are considered major paradigms for selective elimination of superfluous or dysfunctional mitochondria by a specific autophagy process known as mitophagy [64]. We thus questioned the putative contribution of APP-CTFs accumulation to mitophagy. We examined the induction of basal autophagy through the conversion of soluble microtubule-associated protein 1A/1B-light chain 3 (LC3-I) to lipid-bound LC3-II and quantified their expression levels and that of the autophagy substrate SQSTM1/p62 (named p62 hereafter). We specifically quantified LC3 conversion and p62 level in mitochondrial fraction. Our data show a slight but not significant increase of LC3-I but a drastic enhancement of LC3-II level and LC3-II/LC3-I ratio, indicating a basal autophagy induction in APPswe cells (Fig. 5a, b). We further measured mitophagy using LC3-GFP probe and fluorescence microscopy, and confirmed the conversion of LC3-I to LC3-II through aggregation and colocalization of LC3-GFP with HSP60 mitochondrial protein in APPswe cells (Fig. 5e). Paradoxically, the level of p62 remained unchanged (Fig. 5a, b). The increase in LC3-I conversion, LC3-II accumulation, and unchanged p62 levels supports enhanced basal autophagy induction and a blockade in downstream degradation. We then analyzed mitochondrial mitophagy priming occurring through PINK1/Parkin pathway [87]. We observed significantly enhanced levels of Parkin and PINK1 in mitochondrial fraction isolated form APPswe as compared to control cells (Fig. 5a, c). Increased Parkin recruitment to mitochondria was further confirmed by immunofluorescence through its colocalization with mitochondrial HSP60 protein in APPswe cells (Fig. 5f). Accordingly, we also showed a slight increase of mitochondrial localization of phospho-poly-ubiquitin (p-S65-Ub: p-Ub), a PINK1 substrate recruited by Parkin to mitochondria [87] in APPswe cells (Fig. 5f). As control, we analyzed protonophore carbonyl cyanide m-chlorophenyl hydrazone (CCCP)-induced mitophagy and showed enhanced Parkin and p-Ub localization within mitochondria in both control and APPswe-treated cells (Fig. 5f).

Activated mitophagy process is reflected by enhanced degradation of mitochondrial outer and inner membrane and matrix proteins. SDS-PAGE analyses performed on mitochondria-enriched fraction showed increased levels of several mitochondrial proteins (TOMM20, TIMM23, HSP60, and HSP10) in APPswe cells (Fig. 5a, d), suggesting a compromised targeting of dysfunctional mitochondria to lysosomal compartment and/or its defective degradation. Indeed, we observed reduced colocalization of mitochondria with lysosomes in APPswe versus control cells as illustrated through the analyses of the colocalization of mitochondrial Mit-RFP probe with lysosomal LAMP1-GFP probe (Fig. 5g). Furthermore, we used Cox8-EGFP-mCherry mitophagy reporter, [71] (Fig. 5h left) and reported a not statistically different number of cells harboring red mitochondria puncta in APPswe and control cells, thus attesting for defective mitochondrial engulfment in lysosomal acidic compartment (Fig. 5h, i). We validated Cox8-EGFP-mCherry mitophagy reporter by demonstrating enhanced mitophagy (cells harboring red puctae) in control and APPswe cells treated with the iron chelator DFP (deferiprone) (a potent mitophagy inducer in SH-SY5Y cells [1] (Fig. 5h, i), and with a combination of oligomycin A and antimycin A in APPswe cells [1] (Suppl. Figure 8a, online resource).

**Fig. 6:**
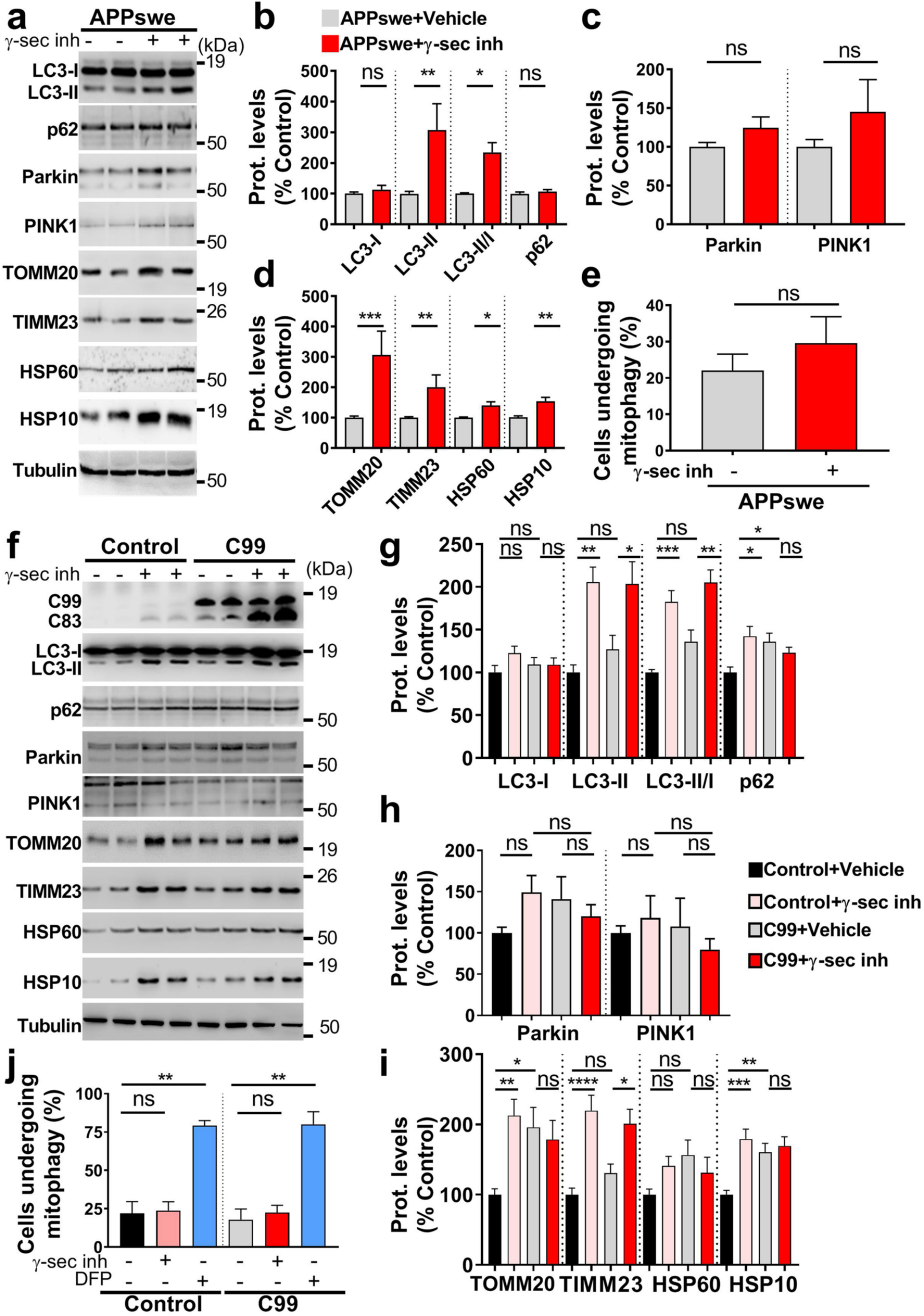
APP-CTFs accumulation/overexpression triggers mitophagy failure in cells. **a** SDS-PAGE of LC3-I and LC3-II, SQSTM1/p62 (p62), Parkin, PINK1, TOMM20, TIMM23, HSP60, and HSP10 in mitochondria-enriched fraction of SH-SY5Y APPswe cells treated with vehicle (−) or with γ-secretase inhibitor (γ-sec inh) ( +) (5 µM, 20 h). **b**–**d** Quantitative graphs of indicated proteins expressed as means ± SEM (*n* = 4–6 in duplicates) versus APPswe cells treated with vehicle (taken as 100%). **e** Graph represents percentage of cells (Means ± SEM of *n* = 130) undergoing mitophagy (analyzed as in Fig. 5h). **f** SDS-PAGE of C99, C83, LC3-I and LC3-II, SQSTM1/p62 (p62), Parkin, PINK1, TOMM20, TIMM23, HSP60, and HSP10 in mitochondria-enriched fraction of SH-SY5Y control and C99 cells as described in Fig. 4a. **g**–**i** Quantitative graphs of indicated proteins expressed as means ± SEM versus control cells (+ vehicle) (taken as 100%) (*n* = 4–5 in duplicates). **j** Graph represents percentage of cells (Means ± SEM of *n* = 70–80) undergoing mitophagy (analyzed as in Fig. 5h). DFP was used a positive control. **a**, **f** Tubulin was used as loading control. **b–e** *P < 0.05, **P < 0.01, ***P < 0.001, and *ns* non-significant versus control using Mann–Whitney test. **g**–**i** *P < 0.05, **P < 0.01, ***P < 0.001, ****P < 0.0001, and *ns* non-significant using one-way ANOVA and Tukey’s multiple comparison post-test. **j** **P < 0.01, and ns: non-significant using Kruskal–Wallis test and Dunn’s multiple comparison post-test

Importantly, pharmacological blockade of γ-secretase in APPswe cells definitely demonstrates that mitophagy failure could be accounted by APP-CTFs accumulation (Fig. 6a–e). Hence, APPswe-treated cells showed a huge increase of LC3-II level and LC3-II/LC3-I ratio, unchanged levels of p62 in mitochondria-enriched fraction (Fig. 6a, b), and enhanced total p62 fluorescence (Suppl. Figure 8b, online resource), attesting for basal autophagy induction and degradation defect. APPswe cells treated with γ-secretase inhibitor also showed defective mitophagy priming with a slight but non-statistically significant increase of Parkin and PINK1 levels in mitochondria (Fig. 6a, c). Unchanged Parkin and p-Ub targeting to mitochondria were further showed by immunofluorescence analyses (Suppl. Figure 8b, online resource). Accordingly, a significant increase of mitochondrial proteins (TOMM20, TIMM23, HSP60, and HSP10) (Fig. 6a, d) in mitochondria-enriched fraction suggested a compromised targeting of dysfunctional mitochondria to lysosomes and their degradation in γ-secretase inhibitor-treated APPswe cells. Indeed, we revealed unchanged colocalization of Mit-RFP probe with LAMP1-GFP probe (Suppl. Figure 8c, online resource), and confirmed unchanged number of mitochondria in fusion with lysosomes using Cox8-EGFP-mCherry probe (Fig. 6e).

Importantly, γ-secretase inhibitor-mediated accumulation of endogenous APP-CTFs was also associated with mitophagy failure in SH-SY5Y control cells characterized by enhanced LC3-II/LC3-I ratio unchanged levels of mitochondrial p62 (Suppl. Figure 9a, b, online resource), enhanced total p62 staining (Suppl. Figure 9d, online resource), unchanged Parkin, and p-Ub (Suppl. Figure 9a, c, and d, online resource), and a significant increase of TIMM23, and HSP10 expressions (Suppl. Figure 9a, c, online resource). We confirmed this mitophagy failure phenotype by showing unchanged colocalization of Mit-RFP probe with LAMP1-GFP probe upon γ-secretase inhibitor treatment (Suppl. Figure 9e, online resource).

Accordingly, we validated mitophagy defect in C99 cellular model and showed a slight but no-statistically significant increase of LC3-II level and LC3-II/LC3-I ratio (Fig. 6f, g) associated with a significant increase of p62 (Fig. 6f, g), unchanged PINK1 and Parkin (Fig. 6f, h), and enhanced TOMM20, and HSP10 expression levels (Fig. 6f–i). Both control and C99-expressing cells treated with γ-secretase inhibitor also showed a mitophagy failure phenotype supported by a significant increase of LC3-II level, and of LC3-II/LC3-I ratio (Fig. 6f, g) associated with unchanged or enhanced p62 (Fig. 6f, g), unchanged PINK1 and Parkin (Fig. 6f, h), enhanced TOMM20, TIMM23, and increase or unchanged HSP10 (Fig. 6f, i). We further showed unchanged levels of Parkin in control and C99-expressing cells treated with γ-secretase inhibitor by immunofluorescence (Suppl. Figure 8d, online resource). Using Cox8-EGFP-mCherry probe, we then demonstrated basal mitophagy defect upon C99 expression or accumulation (Fig. 6j), and revealed as observed in APPswe cells enhanced DFP-induced mitochondria targeting to lysosomal compartment in both control and C99 cells (Fig. 6j).

Interestingly, we also observed unchanged basal autophagy (LC3-I, LC3-II, and p62 levels), mitophagy priming (PINK1, and Parkin), and mitochondrial proteins levels (TOMM20, TIMM23, HSP60, and HSP10) in β-secretase inhibitor-treated APPswe cells, further demonstrating the deleterious effect of accumulated APP-CTFs independently from Aβ on mitophagy failure phenotype (Suppl. Figure 3j, online resource). Overall, these data consistently demonstrate that the accumulation of both endogenous and overexpressed APP-CTFs impaired basal mitophagy.

### Mitochondrial structure alterations in AD mice models are linked to APP-CTFs accumulation but independently of Aβ

**Fig. 7:**
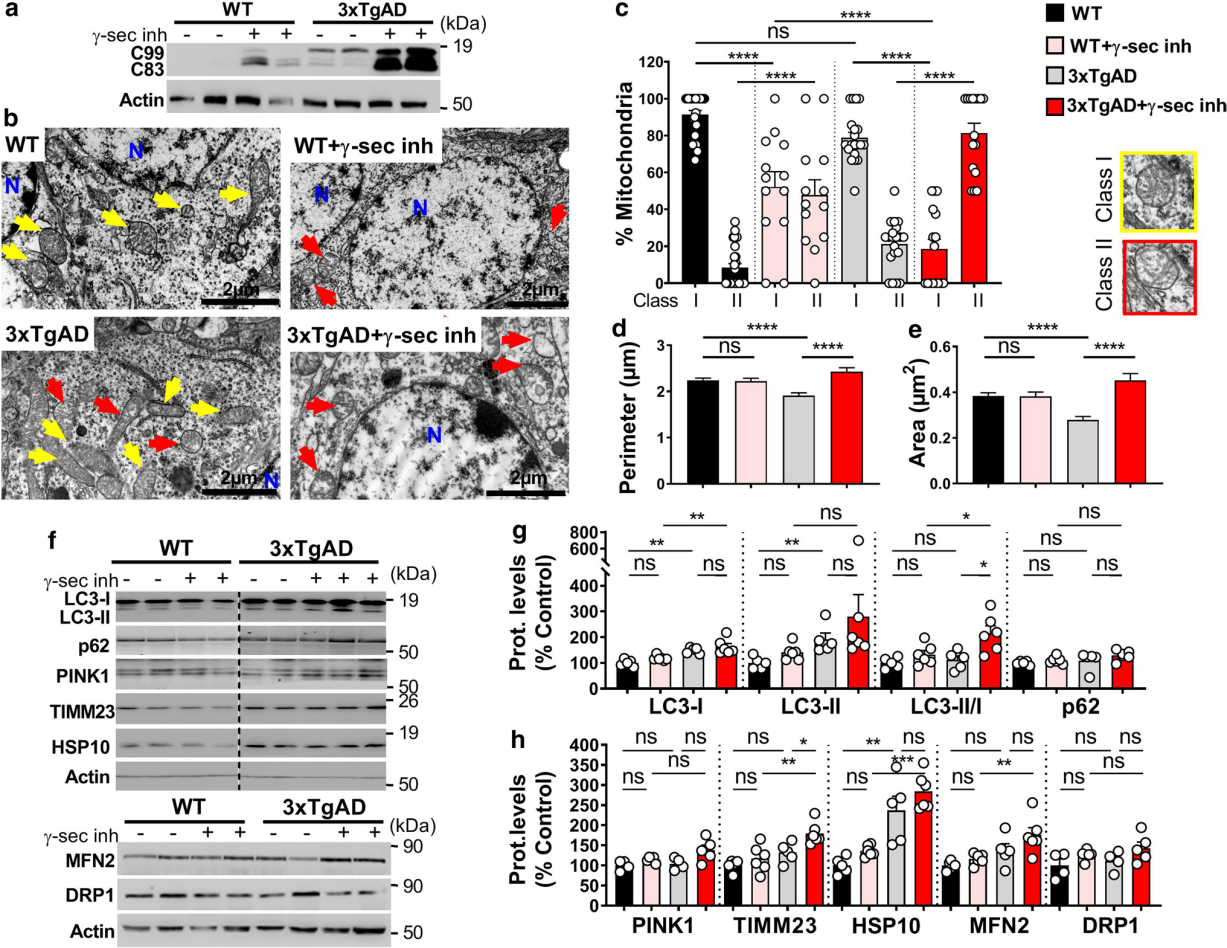
APP-CTFs accumulate in mitochondria and trigger mitochondrial structure alteration and mitophagy failure in 3xTgAD mice. **a** SDS-PAGE of C99 and C83 expression detected using APP-Cter antibody in mitochondria-enriched fraction of hippocampi of 5-month-old wild-type (WT) or 3xTgAD females treated daily by oral gavage (for 1 month) with vehicle (methylcellulose/polysorbate 80) (−) or with γ-secretase inhibitor (γ-sec inh) (30 mg/kg) ( +). **b** Electron microphotographs of neuronal soma of WT and 3xTgAD mice treated as in (**a**). *N* nucleus. Yellow and red arrows indicate mitochondria classes I or II shown in representative images in (**c**, right). Quantitative graphs of mitochondria classes I and II (**c**) and of the means ± SEM of mitochondria perimeter (µm) (**d**) and area (µm^2^) (**e**). **b**–**e** Data were obtained in two mice for each condition (> 20 analyzed field, > 100 of mitochondria). **f** SDS-PAGE of LC3-I and LC3-II, SQSTM1/p62 (p62), PINK1, TIMM23, HSP10, MFN2, and DRP1 in mitochondria-enriched fraction of hippocampi of WT and 3xTgAD mice treated as in (**a**). **g**, **h** Quantitative graphs of indicated proteins expressed as means ± SEM versus WT mice (treated with vehicle) (taken as 100%) (WT + veh, *n* = 4–5; WT + γ-sec inh, *n* = 5–6; 3xTgAD + veh, *n* = 4–5; 3xTgAD + γ-sec inh, *n* = 6). **a**, **f** Actin was used as loading control. **c**–**e** ****P < 0.0001, and *ns* non-significant using Kruskal–Wallis test and Dunn’s multiple comparison post-test. **g**, **h** *P < 0.05, **P < 0.01, ***P < 0.001, and ns: non-significant using one-way ANOVA and Tukey’s multiple comparison post-test

**Fig. 8:**
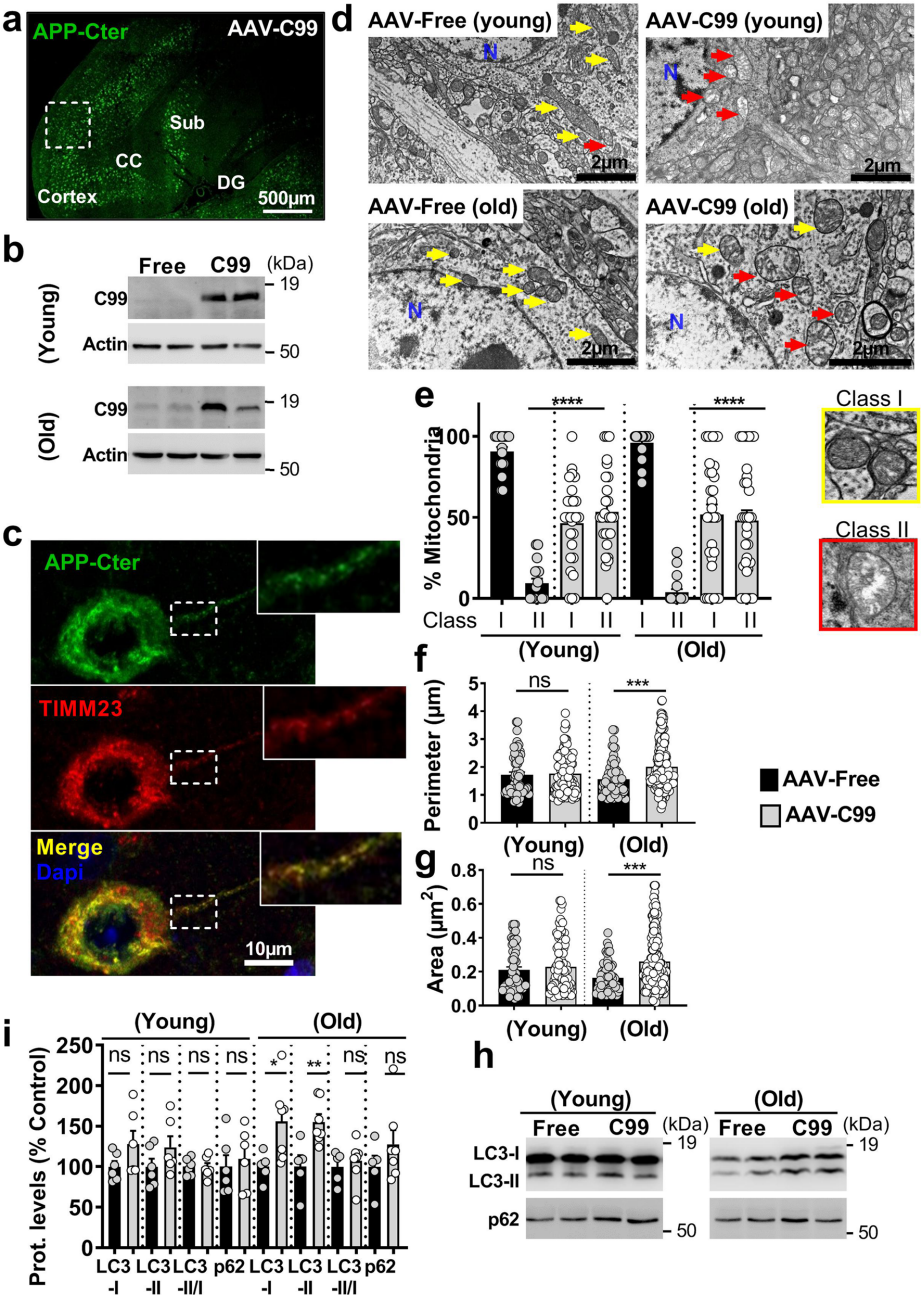
Adeno-associated viral (AAV)-mediated expression of C99 in wild-type mice leads to APP-CTFs accumulation in mitochondria and triggers mitochondrial structure alteration and mitophagy failure phenotype. **a** Brain section of AAV-C99 injected (12-month-old) mice immunostained with APP-Cter antibody. Brain regions are depicted as cortex, corpus callosum (CC), subiculum (sub), and dentate gyrus (DG). Boxed cortex area represents region analyzed by electron microscopy. Scale bar represent 500 µm.** b** SDS-PAGE of C99 expression detected using APP-Cter antibody in mitochondria-enriched fraction of brains of AAV-Free (Free) or AAV-C99 (C99) injected mice aged 2–3 months (young) or 12 months (old). Actin was used as loading control. **c** Immunostaining of C99 neuronal expression in AAV-C99-injected mice (12 month-old) using APP-Cter antibody (green) and of mitochondria using TIMM23 antibody (red). Nuclei were labeled with DAPI. Higher magnification of boxed area represents axonal region. Colocalization of C99 and TIMM23 (yellow merged signal) is observed in soma and axon. Scale bar represent 10 µm. **d** Electron microphotographs of neuronal soma of young and old AAV-free and AAV-C99 mice. *N* nucleus. Yellow and red arrows indicate mitochondria class I or class II respectively shown in representative images in (**e** right). **e–g** Quantitative graphs of mitochondria classes I and II (**e**) and of the means ± SEM of mitochondria perimeter (µm) (**f**), and area (µm^2^) (**g**). **d**–**g** Data were obtained in 2–3 different mice in each condition (> 20 analyzed field, > 100 mitochondria). **h** SDS-PAGE of LC3-I and LC3-II, and SQSTM1/p62 (p62) in mitochondria-enriched fraction of brains of young and old AAV-free and AAV-C99 mice. **i** Quantitative graphs of indicated proteins expressed as means ± SEM versus AAV-free mice (taken as 100%) (AAV-free mice young *n* = 6, old, *n* = 5; and AAV-C99 mice young *n* = 6, and old, *n* = 7)**. e**–**g** ***P < 0.001, ****P < 0.0001, and ns: non-significant using Kruskal–Wallis test and Dunn’s multiple comparison post-test. **i** *P < 0.05, **P < 0.01, and *ns* non-significant versus respective AAV-free versus AAV-free mice using Mann–Whitney test

We already reported early and progressive APP-CTFs accumulation in the subiculum of 3xTgAD mice model [43], and showed that in vivo treatment of young 3xTgAD mice with the γ-secretase inhibitor enhances APP-CTFs accumulation and triggers massive increases in endosome autophagic lysosomes (EAL) accumulation [45]. We took advantage of this model to examine the impact of APP-CTFs accumulation on mitochondrial structure and mitophagy in vivo. Young mice (aged 5 months) were treated daily for 1 month with γ-secretase inhibitor and analyzed for APP-CTFs’ expression/accumulation in mitochondria-enriched fraction. We first show the presence of APP-CTFs in mitochondria of 3xTgAD but not WT mice hippocampi (Fig. 7a). γ-secretase inhibitor unravels APP-CTFs accumulation in WT mice and drastically enhanced it in 3xTgAD mice (Fig. 7a). Mitochondria morphology and size were then analyzed by electron microscopy in the subiculum area of WT and 3xTgAD mice treated with vehicle or with γ-secretase inhibitor (Fig. 7b-e). We constrained our classification in vivo to two mitochondria classes, where class I corresponds to mitochondria with uniform matrix filled with dense packed regular distributed cristae, and class II corresponds to mitochondria showing morphological abnormalities, ranging from focal loss of cristae with empty spaces to severe loss of cristae and matrix (corresponding to mitochondria classes II and III in cells). We did not observe swollen mitochondria class IV in vivo. We first show that vehicle-treated WT and 3xTgAD mice harbor 91% and 79% of class I mitochondria, respectively (Fig. 7c and suppl. Table 3, online resource). Thus, 3xTgAD mice did not show noticeable early alteration of mitochondria cristae shape. However, we observed that mitochondria of 3xTgAD mice harbor a significant reduction of perimeter and area as compared to wild-type mice (Fig. 7d, e and suppl. Table 3, online resource). Importantly, the treatment with γ-secretase inhibitor dramatically and significantly lowered the percentage of class I mitochondria to 19%, and 52%, and enhanced the percentage of class II mitochondria that reached 81% and 48% in 3xTgAD and WT mice respectively (Fig. 7b, c and suppl. Table 3, online resource). γ-secretase inhibitor treatment also enhanced mitochondria perimeter and area in 3xTgAD mice without affecting that of WT mice (Fig. 7d, e and suppl. Table 3, online resource). To confirm that C99 accumulation per se also impacts mitochondria morphology and size in vivo; we compared young (aged 2–3 months) and old (aged 12 months) adeno-associated-virus (AAV)-C99 injected mice previously described [45]. Immunofluorescence analyses using APP-Cter antibody showed intraneuronal expression of C99 in the cortex and the subiculum (Sub) and dentate gyrus (DG) regions of the hippocampus of AAV-C99 injected mice (Fig. 8a). C99 expression was detectable in mitochondrial fractions isolated from both young and old AAV-C99 mice brains (Fig. 8b) and only faintly detectable in AAV-Free-injected mice (Fig. 8b). This observation was corroborated by demonstrating the colocalization of C99 (using APP-Cter antibody) with mitochondrial TIMM23 protein (Fig. 8c). We analyzed mitochondrial morphology in the cell body of cortical neurons and revealed a large number of mitochondria class I in young and old control mice (91% and 96% respectively). In contrast, we noticed a reduction of mitochondria class I in both young and old AAV-C99 injected mice (47% and 52%, respectively) with concomitant increases in class II mitochondria (53% and 48%, respectively) (Fig. 8d, e and suppl. Table 3, online resource). In AAV-C99-injected mice, C99 is also expressed in the hippocampus (Fig. 8a and suppl. Figure 10a, online resource), and triggers reduced class I mitochondria in the cell body of hippocampal neurons (Suppl. Figure 10b, c, online resource). Interestingly, we observed unchanged mitochondria size (area and perimeter) in AAV-C99 versus AAV-Free young mice (Suppl. Figure 10b, d, and e, online resource), but significantly increased size in AAV-C99 old mice as compared to their age-matched control mice (Fig. 8f, g).

The similar data obtained in γ-secretase inhibitor-treated 3xTgAD mice (Fig. 7a–e) and AAV-C99 mice (Fig. 8a–g and suppl. Figure 10b-e, online resource) fully demonstrate that APP-CTFs accumulate in mitochondria and induce early and age-dependent mitochondrial structure alterations.

### Basal mitophagy failure in AD mice models is associated with APP-CTFs accumulation

The analysis of mitophagy marker expressions showed enhanced mitochondrial LC3-I and LC3-II and unchanged p62 levels in 3xTgAD mice than in WT mice reflecting both basal autophagy induction (conversion of LC3-I- to LC3II) and impairment of autophagy degradation (LC3-I, LC3-II accumulation and unchanged p62 level) (Fig. 7f, g). Since at 5-month age, 3xTgAD mice display high APP-CTFs levels, while Aβ remained barely detectable [11], we may assume that mitophagy failure is rather associated with APP-CTFs accumulation. Corroborating this statement, we observed increase of LC3-I level and of LC3-II/LC3-I ratio, unchanged p62 level in 3xTg-AD versus WT mice treated with γ-secretase inhibitor (Fig. 7f, g). Accordingly, old but not young AAV-C99-injected mice showed basal autophagy induction and defective autophagy degradation (LC3-I, LC3-II accumulation and unchanged p62 level) (Fig. 8h, i). In agreement with defective degradation of dysfunctional mitochondria, we noticed unchanged level of the mitophagy priming protein PINK1 in mitochondria fraction of vehicle- or with γ-secretase inhibitor-treated 3xTgAD mice (Fig. 7f, h), and enhanced HSP10 protein level in 3xTg-AD mice versus WT mice (Fig. 7f, h), and observed a significant enhancement of TIMM23, HSP10, and MFN2 protein levels in 3xTg-AD versus WT mice treated with γ-secretase inhibitor (Fig. 7f, h). These data fully demonstrate that APP-CTFs accumulation triggers mitophagy failure in vivo.

### Mitochondria structure alterations and mitophagy are differently impacted by late-stage Aβ and APP-CTFs accumulation in AD mice models

**Fig. 9:**
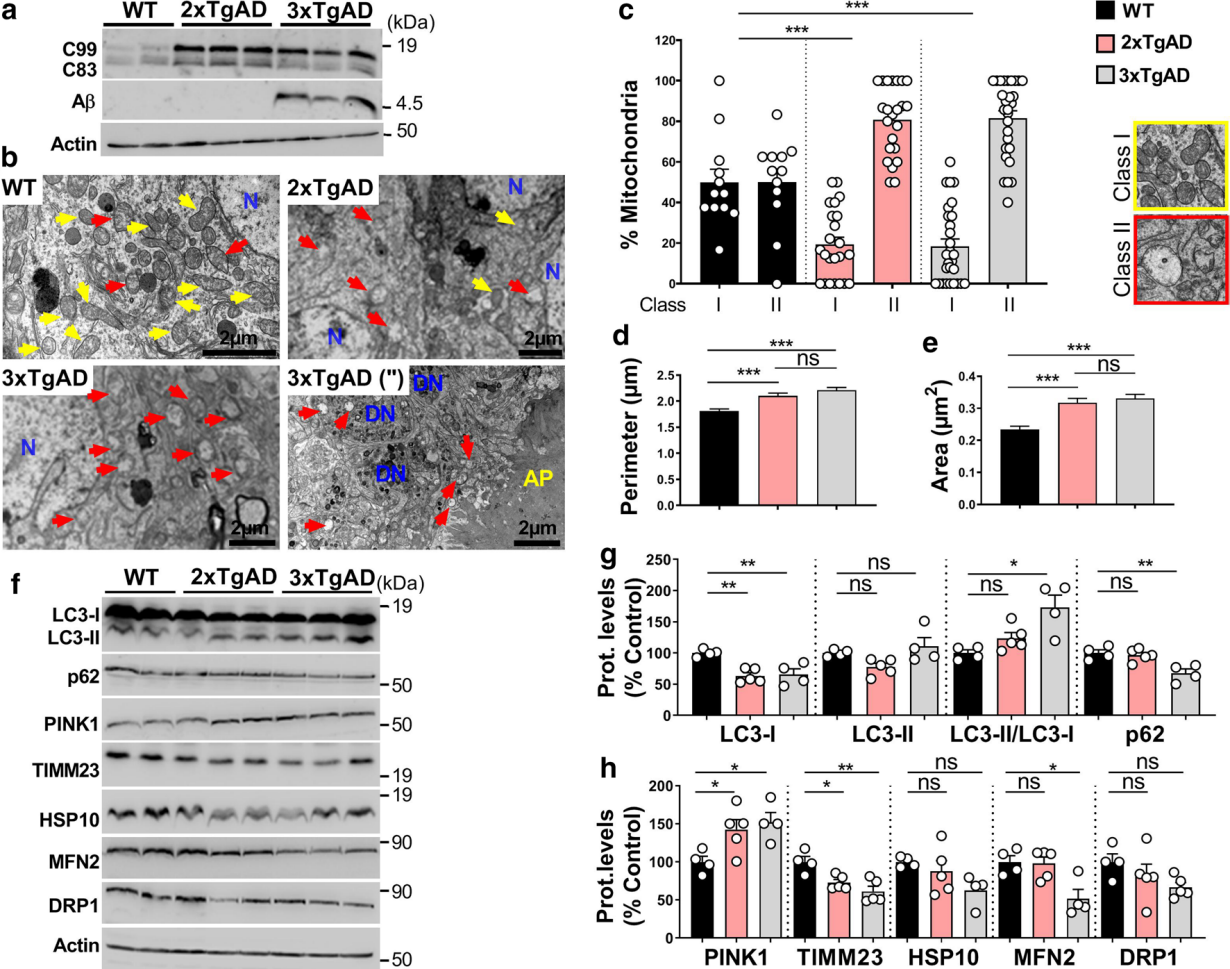
Both APP-CTFs and Aβ contribute to mitochondrial structure and mitophagy alterations in old AD mice models. **a** SDS-PAGE of C99, C83, and Aβ expression in mitochondria-enriched fraction of hippocampi of 17 month-old wild-type (WT), 2xTgAD, and 3xTgAD males detected using 6E10 antibody. **b** Electron microphotographs of neuronal soma of WT, 2xTgAD, and 3xTgAD mice. 3xTgAD (") electron microphotograph represents amyloid plaque (AP) and dystrophic neuritis (DN). *N* nucleus. Yellow and red arrows indicate mitochondria classes I or II shown in representative images in (**c** right). **c-e** Quantitative graphs of mitochondria classes I, and II (**c**) and of the means ± SEM of mitochondria perimeter (µm) (**d**), and area (µm^2^) (**e**). **b**–**e** Data were obtained in two mice for each condition (> 20 analyzed field, > 100 mitochondria). **f** SDS-PAGE of LC3-I and LC3-II, SQSTM1/p62 (p62), PINK1, TIMM23, HSP10, MFN2, and DRP1 in mitochondria-enriched fraction of hippocampi of WT, 2xTgAD, and 3xTgAD mice. **a**, **f** Actin was used as loading control. **g, h** Quantitative graphs of indicated proteins expressed as means ± SEM versus WT mice (taken as 100%) (WT, *n* = 4; 2xTgAD, *n* = 5; 3xTgAD, *n* = 5). **c-e** ***P < 0.001, and *ns* non-significant using Kruskal–Wallis test and Dunn’s multiple comparison post-test. **g**, **h** *P < 0.05, **P < 0.01, and *ns* non-significant using one-way ANOVA and Tukey’s multiple comparison post-test

To delineate the respective contribution of APP-CTFs and Aβ to mitochondrial structure and mitophagy defects, we compared 3xTg-AD and 2xTg-AD mice. Thus, while both AD models accumulate APP-CTFs similarly, only 3xTg-AD animals produce Aβ (at late age) that remains always barely detectable in 2xTg-AD mice [11]. In fact, we confirmed similar APP-CTFs accumulation in mitochondria-enriched fractions from both 2xTg-AD and 3xTg-AD and noticeable absence of Aβ peptide in 2xTgAD mice (Fig. 9a). We also confirmed the presence of large amyloid plaques (AP) surrounded by dystrophic neurites (DN) in the subiculum of 3xTg-AD mice but not in 2xTg-AD mice [Fig. 9b, image 3xTg-AD (”)]. Interestingly, electron microscopy analyses revealed that while old WT mice display equal proportions of classes I and II mitochondria, both old 2xTgAD and 3xTgAD mice display a higher population of class II mitochondria (81% and 82%, respectively) (Fig. 9c and suppl. Table 3, online resource) and significant increases in mitochondria perimeter (Fig. 9d and suppl. Table 3, online resource) and area (Fig. 9e and suppl. Table 3, online resource). These data demonstrate that mitochondria structure alteration in old mice is triggered by the accumulation of APP-CTFs independently of Aβ and pTau (accumulating in old 3xTg-AD but not in 2xTg-AD mice [11]) (Fig. 9b–e).

Mitophagy analyses revealed in old 3xTgAD mice reduced LC3-I level, unchanged LC3-II level, and enhanced LC3-II/LC3-I ratio (Fig. 9f, g). In accordance, with active mitophagy, old 3xTgAD mice showed reduced p62 (Fig. 9f, g), enhanced PINK1 (Fig. 9f, h), and reduced levels of TIMM23 and MFN2 (Fig. 9f, h). We also noticed reduction trends of HSP10 and DRP1 proteins, but that did not reach statistical significance (Fig. 9f, h). Unlike 3xTgAD mice, 2xTgAD mice harbor a mitigate mitophagy phenotype showing reduced LC3-I, unchanged LC3-II levels and LC3-II/LC3-I ratio (Fig. 9f, g), unchanged p62 (Fig. 9f, g), and enhanced PINK1 and a slight reduction of TIMM23 but not of HSP10, MFN2, or DRP1 (Fig. 9f–h). Altogether, these results pinpointed a late-stage contribution of Aβ and pTau to mitophagy induction in old 3xTgAD mice (Fig. 9f–h), while early APP-CTFs accumulation accounts for impaired mitophagy in young 3xTgAD (Fig. 7f–h) and old 2xTgAD mice (Fig. 9f–h). The specific contribution of Aβ versus pTau to mitophagy activation in old 3xTg-AD mice needs a dedicated study.

### Correlation between basal mitophagy failure and APP-CTFs accumulation in human AD brains

**Fig. 10:**
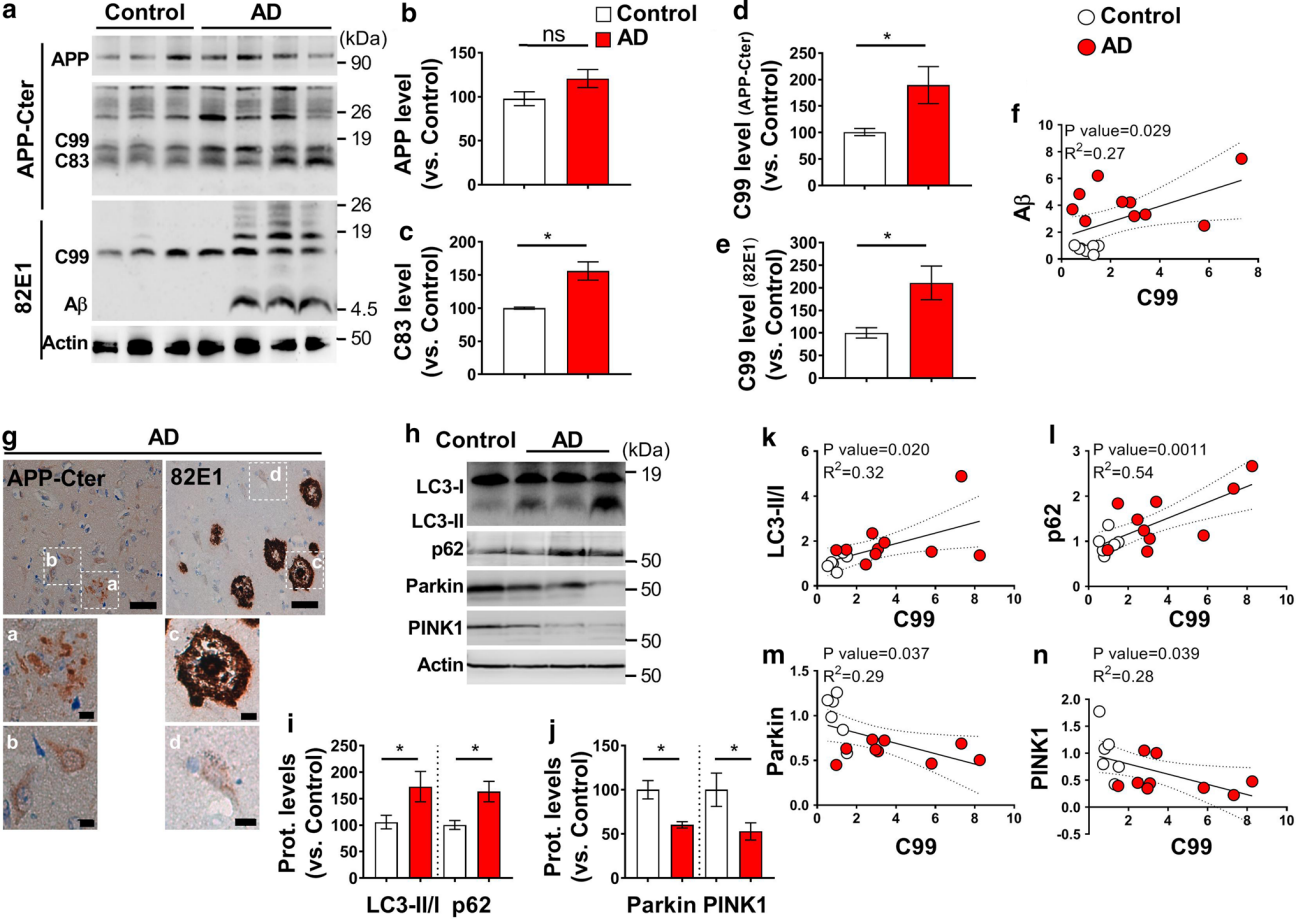
APP-CTFs accumulate in SAD brains and are associated with basal mitophagy failure. **a** SDS-PAGE of full-length APP (APP), C99, C83, and Aβ expression in mitochondria-enriched fraction of temporal lobe of human control (*n* = 6–7) and AD brains (*n* = 10–11) (patients information in suppl. Table 1, online resource), revealed using APP-Cter (upper panel), and 82E1 (lower panel) antibodies. **b**–**e** Quantitative graphs of indicated proteins expressed as means ± SEM versus controls (taken as 100%). **f** Correlation plots between Aβ and C99 levels including controls (white dots), and AD (red dots) brain samples. **g** Immunohistochemical staining of a human AD brain (T1 region of the temporal lobe) with APP-Cter and 82E1 antibodies. Higher magnification of boxed area represents extracellular aggregates and plaques (**a**, **c**), and intra-neuronal signal (**b**,** d**). Scale bars represent 50 µm and 10 µm. Nuclei were stained with cresyl violet. **h**–**j** SDS-PAGE of LC3-I and LC3-II, SQSTM1/p62 (p62), Parkin, and PINK1 in mitochondria-enriched fraction of temporal lobe of human control (**h**), and quantitative graphs of indicated proteins (mean ± SEM versus control taken as 100%) of control (*n* = 6–8) and AD brains (*n* = 9–14) (patients information in suppl. Table 1, online resource) (**i**, **j**). **a**, **h** Actin was used as loading control. **k**–**n** Correlation plots between C99 and LC3-II/I (**k**), p62 (**l**), Parkin (**m**), and PINK1 (**n**) levels in controls (white dots), and AD (red dots) brains. **f**, **k**–**n** Linear regression was used to determine *P* and goodness of fit (*R*^2^) values. **b**–**e**, **i**, **j** *P < 0.05, and *ns* non-significant versus control using Mann–Whitney test

We lastly questioned whether APP-CTFs accumulate in human SAD brains and investigated the potential correlation between APP-CTFs levels in mitochondria and mitophagy markers. The presence and accumulation levels of APP-CTFs were explored in mitochondria-enriched fraction of a large human SAD brains cohort (patients’ information in suppl. Table 1, online resource). We observed unchanged level of full-length APP (Fig. 10a, b), and enhanced C83 and C99 expressions (Fig. 10a, c–e) in AD brains using the two sets of antibodies APP-Cter and 82E1 (directed to the first and free aa residue of Aβ and C99) (Fig. 10a, d, e). In this cohort, we also revealed a positive and significant correlation between C99 level and total Aβ load (Fig. 10f). Using immunohistochemistry and antigen retrieval approaches [16], we revealed in AD brain slices (patients information in suppl. Table 1, online resource) intracellular APP-CTFs and Aβ membranous-like signal (Fig. 10g, inserts b, d), in parallel to extracellular APP-CTFs’ aggregates and focal amyloid plaques, respectively, detected with APP-Cter and 82E1 antibodies (Fig. 10g, inserts a, c). We investigated in the same brain cohort mitophagy markers (LC3-I, LC3-II, P62, Parkin, and PINK1) expression in mitochondria-enriched fractions (Fig. 10h–n). Interestingly, we showed a significant increase of LC3-II/LC3-I and enhanced p62 level in AD brains as compared to controls (Fig. 10h, i). Supporting mitophagy priming failure, we also revealed a consistent reduction of PINK1 and Parkin proteins (Fig. 10h, j). Importantly, expression of all these markers significantly correlated with mitochondrial C99 level (Fig. 10k–n). Since our AD cohort shows elevated expression levels of Aβ (Fig. 10a, f) and pTau (Suppl. Figure 11e, online resource), we also analyzed the potential correlation of mitophagy markers expression with Aβ and pTau levels. Intriguingly, we noticed a significant negative correlation of total Aβ load with Parkin (Suppl. Figure 11c, online resource), but not with LC3-II/LC3-I, p62 and PINK1 (Suppl. Figure 11a, b, and d, online resource). We also observed a positive correlation of pTau with LC3-II/LC3-I ratio (Suppl. Figure 11f, online resource), but not with the other markers (Suppl. Figure 11 g-i, online resource). These data are in agreement with previous studies, showing that Parkin trigger intracellular Aβ42 clearance [12] and the colocalization of pTau with LC3 in FAD brains [65]. Overall, these data firmly emphasize a strong correlation between APP-CTFs accumulation and defective mitophagy phenotype in human AD brains, thus supporting our findings in various cellular and mice AD models.

## Discussion

We report that APP-CTFs accumulation triggers mitochondrial dysfunctions and mitophagy failure in an Aβ-independent manner in various AD study models. This was demonstrated by studying a familial AD mutation known to enhance APP-CTFs production and by pharmacological blockade of γ-secretase that increases APP-CTFs recovery and impairs Aβ and AICD productions as well as by the study of models overexpressing C99 fragment. Consistent alterations were observed in cellular and in vivo mice models as well as in SAD-affected brains.

Several studies show that the β-secretase-derived APP-CTF is neurotoxic perse [38, 45, 58, 85]. Furthermore, transgenic mice expressing C99 fragment (C100-Tg) or stereotaxically injected with purified APP-βCTF (C100) into the brain show neuronal degeneration in the hippocampus, cortical atrophy, astrogliosis, reduced LTP, and behavioral deficits [6, 52–54]. These phenotypes have also been observed in wild-type or transgenic APP mice models after treatment with γ-secretase inhibitors [7, 52], and were amplified in C100-Tg mice harboring familial AD mutation [10]. Accordingly, we reported early, age-dependent, and hippocampus-specific accumulation of C99 in 3xTgAD mice model [43], and demonstrated that C99 accumulation in mice (rather than Aβ) is associated with synaptic alterations, and apathy-like behavior [11, 45]. Our data led us to envision that APP-CTFs accumulation could trigger mitochondrial structure, function, and mitophagy alterations that are consistently observed in AD-affected brains, and thereby could contribute to AD development or progression. This hypothesis is supported by evidence that mitochondria structure, function, and position directly impact neuronal functioning, thus controlling synaptic plasticity, learning and memory [72].

First, we revealed that APP-CTFs accumulate in mitochondria-enriched fraction and colocalize with mitochondrial proteins in cells and mice AD models. This agrees well with previous studies, demonstrating that APP is associated with mitochondria in AD-affected neurons [2, 3], and that APP catabolites (i.e., Aβ, C99, and AICD) are present in mitochondria [15, 20, 21, 60, 61, 74]. Supporting APP targeting to mitochondria, mutants lacking a region that encompasses a 5-aa trans-membrane (TM) and 7-aa juxta-membrane (JM) regions exhibit decreased mitochondrial localization [81]. Our laboratory and another also recently demonstrated the presence of APP and its derived fragments in mitochondria-associated membranes (MAMs) in sporadic and familial cellular and mice AD models [20, 63]. We also unraveled the presence and activity of β- and γ-secretases in MAMs, thus allowing APP processing in this microdomain [20]. Importantly, we demonstrate herein that APP-CTFs accumulation also occurs in mitochondria of human AD brains in agreement with previous studies showing enhanced BACE1 and β-secretase-derived APP-CTF levels in autosomal-dominant AD human brain total extracts [25, 62, 66].

Previous studies showed Aβ interaction with several mitochondrial proteins [82], and that Aβ triggers reduced activity of respiratory chain complexes [8, 24, 40]. In agreement with mitochondrial OXPHOS dysfunction and mitochondrial swelling (depicted as mitochondria type IV in cells), we noticed enhanced STS-induced caspase-3 activation in APPswe-expressing cells that was reversed by both β-secretase and several γ-secretase inhibitors. This demonstrates that caspase-3 activation in APPswe cells treated with STS is mostly contributed by Aβ but not by APP-CTFs. Conversely, we did not evidence STS-induced caspase-3 activation in C99-expressing cells likely attributable to different Aβ levels/subtypes produced in APPswe cells (Aβ40 and Aβ42 [59]) versus C99-expressing models (Aβ40 [45]). Interestingly, mitochondrial ROS levels were enhanced in APPswe and C99-expressing cells, and potentiated by γ-secretase inhibitor in APPswe-expressing cells. Supporting this observation, APP-CTFs were also shown to enhance nitric oxide level in glial cells [67]. Several studies also reported that mitochondrial Aβ triggers ROS overproduction likely through complex I deficiency and/or interaction with antioxidant enzymes and that ROS increases in turn Aβ production [8, 48, 75]. These data led us to hypothesize that APP-CTFs, ROS, and Aβ may act in concert to accelerate disease progression.

We analyzed in depth mitochondrial structure in cellular and mice AD models using electron microscopy and high-resolution 3D imaging. We first described in APPswe cells large mitochondria with huge cristae disorganization (Fig. 1a, f and suppl. Table 3, online resource). Intriguingly, we revealed that γ-secretase inhibition-mediated APP-CTFs accumulation triggers a specific mitochondrial altered phenotype in cells. In contrast, β-secretase inhibition triggers a recovering of mitochondria class I morphology and a reduction of mitochondria classes II, III, and IV (Suppl. Figure 3d, e, online resource, and suppl. Table 3, online resource). These findings pinpointed the contribution of both Aβ and APP-CTFs accumulation to mitochondrial structure alterations observed in APPswe-expressing cells. Importantly, we also unraveled early mitochondrial morphology alteration, in vivo, in young 3xTgAD mice (5-month-old harboring APP-CTFs accumulation and not-detectable Aβ level) and further demonstrated an APP-CTFs-dependent (Aβ-independent) mitochondrial structure alteration in 3xTgAD mice treated with γ-secretase inhibitor (Fig. 7b, e and suppl. Table 3, online resource). Age- and APP-CTFs accumulation-dependent mitochondrial alterations were then confirmed in old AAV-C99-injected mice showing alteration of both morphology (cristae alteration) and increased size as compared to young AAV-C99-injected mice (Fig. 8d, g and suppl. Table 3, online resource). It is important to note that the alterations of mitochondria size (area and perimeter) were different between cellular models and AD mice brains (Fig. 7). These apparent discrepancies could be linked to APP metabolite levels/kinetic accumulation in these models. Another fundamental finding was obtained in aged (17 months) 2xTgAD (accumulating APP-CTFs but not Aβ) harboring similar mitochondrial structure alterations as observed in old 3xTgAD mice, thus further confirming that APP-CTFs accumulation triggers mitochondrial structure alterations independently of Aβ (Fig. 9 and suppl. Table 3, online resource). In accord with our findings, changes in mitochondrial morphology were reported in the hippocampus of APP/PS1 dE9 mice at age 3 months before the onset of memory decline and Aβ deposition occurring by 6–9 months [83]. However, our data and others revealed that these mitochondrial structure phenotypes were not corroborated by consistent modulation of mitochondrial fission and fusion protein expression. Several studies reported imbalanced mitochondria fission/fusion in AD study models (cells expressing APP familial mutations or treated with Aβ, primary neurons from AD mice) and in human-derived fibroblasts and brains [30]. However, while mitochondrial fragmentation and abnormal distribution were generally described, the underlying molecular mechanisms are inconsistent between models [30, 49, 79, 88]. The variability in the exact molecular mechanisms underlying imbalanced fission/fusion observed in AD may emphasize the complexity of mitochondrial dynamics at different stages of the disease.

Inadequate mitophagy capacity in eliminating damaged mitochondria in AD-affected neurons likely contributes to AD development and/or progression. Recent datasets’ analyses from healthy individuals and AD patients revealed a strong correlation between mitophagy and AD [76]. Several studies also highlighted defective mitophagy in vitro and in vivo linked to Aβ and pTau [17, 35, 50, 69]. We demonstrate herein defective mitophagy phenotypes in both cellular and AD mice models. Importantly, the use of APPswe and C99 cells treated with γ-secretase inhibitor supported the link between APP-CTFs accumulation and mitophagy failure phenotype that we further confirmed by the reduced/or unchanged colocalization/targeting of mitochondria with/to lysosomes. We also delineated that mitophagy blockade occurs at different steps of the process in our cellular models. Thus, while PINK1-Parkin pathway was activated in APPswe-expressing cells, it was abrogated in C99-expressing cells and upon γ-secretase inhibitor treatment-mediated APP-CTFs accumulation. Importantly, we confirmed defective mitophagy priming in young 3xTgAD mice treated with γ-secretase inhibitor. All together, our data suggest “abortion” of mitophagic process in both cellular and AD mice models, since we observed basal autophagy induction and inefficient mitochondrial protein degradation.

Concerning the molecular mechanisms, we recently demonstrated a transcriptional regulation of PINK1 by AICD in a PS1-dependent manner [31] and confirmed herein enhanced PINK1 expression and mitochondrial recruitment of Parkin in APPswe cells. PINK1 stabilization to mitochondria may likely also be attributable to mitochondrial potential depolarization observed in APPswe cells. Consistently, unchanged PINK1 expression in APPswe cells treated with γ-secretase inhibitor and in C99 cells is likely linked to: (1) unchanged or barely detectable AICD levels; and/or (2) unchanged or enhanced mitochondrial membrane potential observed in these cellular models. Accordingly, we also observed unchanged Parkin level in both APPswe cells treated with γ-secretase inhibitor and in C99 cells. We may also consider other molecular mechanisms impacting PINK1 expression in AD. Indeed, we recently reported that nuclear p53-mediated repression of autophagy occurs through PINK1 gene transcriptional down regulation [32], and it is known that p53 levels are enhanced in AD brains [37]. Intriguingly, we revealed late-stage mitophagy activation (LC3-II activation/accumulation, reduced p62, and mitochondrial protein degradation) in old 3xTgAD mice that was larger as compared to 2xTgAD mice likely pointing-out the contribution of late-stage Aβ accumulation and enhanced pTau to excessive mitochondrial degradation in AD. Corroborating this late-stage mitophagy activation, we also showed increased PINK1expression in aged 2xTgAD and 3xTgAD (as we previously reported [31]).

Finally in our SAD cohort, we demonstrated a defective mitophagy phenotype (enhanced LC3-II/LC3-I and p62 and a reduction of Parkin and PINK1 expression) significantly correlating with enhanced C99 levels. Importantly, our data revealed a stronger correlation of mitophagy failure molecular signature with C99 as compared to Aβ or pTau. Clustered AV-like organelles and abnormal mitochondria with swollen shape and loss of cristae integrity were observed in neuronal perikarya of AD patient brains [86]. Impairment of mitophagy has also been observed in SAD fibroblasts due to diminished Parkin recruitment to mitochondria, accumulating depolarized mitochondria and PINK1 [51]. Reduced soluble Parkin and enhanced insoluble Parkin colocalizing with Aβ were also reported in AD brains [47]. Inconsistently, while Martin-Maestro et al. showed enhanced PINK1, Parkin, and TOMM20 expressions [51], Ye et al. noticed a reduced cytosolic and increased mitochondrial Parkin levels in AD brains [86]. These discrepancies rather pointed out the complexity of a unique molecular pathway underlying mitophagy dysfunctions in human AD brains.

Our findings support a notion that enhanced LC3-II activation/accumulation associated with unchanged p62, inconsistent recruitment of PINK1/Parkin to mitochondria and elevated levels of mitochondrial proteins could be attributed to both induction of basal autophagy and defective clearance of autophagic substrates due to increased mitochondrial stress or dysfunction in AD neurons. Importantly, we and others demonstrated that APP-CTFs accumulation is associated with lysosomal–autophagic pathology in vitro and in vivo [36, 39, 41, 45, 53, 77]. Of most interest, a recent work based on either isogenic knockin human iPSCs or monogenic iPSCs carrying mutations on APP or PS1 demonstrated a C99-mediated and Aβ-independent alterations of the endolysosomal network [36, 39, 41, 45, 53, 77]. This importantly indicated that these phenotypic alterations could not be accounted for APP-CTFs overload linked to its overexpression. Furthermore, our laboratory and others also unraveled an amplifying loop where lysosomal–autophagic dysfunction trigger lysosomal APP-CTFs accumulation [5, 33, 45]. A recent study reported that mutated PS2 impairs autophagy by causing a block in the degradative flux at the level of the autophagosome–lysosome fusion step [27]. Accordingly, reduction of NRBF2 (nuclear receptor-binding factor 2) expression, a key component and regulator of the autophagy-associated PIK3C3-containing phosphatidylinositol 3-kinase (PtdIns3K) complex, has been involved in APP-CTFs accumulation and defective autophagy [84]. All together, these evidences suggest that APP-CTFs accumulation triggers mitophagy alteration by interfering with different steps in mitophagic process likely occurring along stages of the disease development.

Several recent data support the relevance of enhancing mitochondrial proteostasis to delay AD pathogenesis. Notably, overexpression of Parkin improves its targeting to mitochondria and potentiates autophagic vesicle synthesis, and decreases the level of Aβ42 and pTau [47, 51]. Accordingly, targeting proteostasis increases the fitness and lifespan of worms and reduces amyloid aggregation in cells, worms, and in transgenic mouse models of AD [76]. The contribution of Aβ-to-AD development has been questioned these last years, since all Aβ-centered therapeutic strategies (i.e., immunotherapy, or β- and γ-secretases inhibitors) failed to interfere with cognitive decline [78]. Our study highlights, at least in part, the proven risk of using γ-secretase inhibitors as a therapeutic strategy in AD. It conversely unravels the potential interest of circumscribing APP-CTFs accumulation to recover mitochondrial structure and functions in AD. Alternatively, pharmacologically and genetically targeting mitochondrial function and mitophagy may also be a valuable strategy to treat or alleviate AD pathogenesis.

## Electronic supplementary material

Below is the link to the electronic supplementary material.

Supplementary file1 (PDF 1716 kb)
