# Supplementary material for: Accumulation of amyloid precursor protein C-terminal fragments triggers mitochondrial structure, function, and mitophagy defects in Alzheimer’s disease models and human brains
Source: Acta Neuropathol. 2020 Oct 20;141(1):39–65. doi: 10.1007/s00401-020-02234-7 (PMC7785558; doi:10.1007/s00401-020-02234-7)
Supplement: Supplementary file 1 — Supplementary file1 (PDF 1716 kb) [file 401_2020_2234_MOESM1_ESM.pdf]

Supplementary Materials and methods

Cells and treatments

Wild-type MEF cells or double invalidated for APP and APLP2 were provided by Dr U. Muller [2] MEF cell lines were cultured in Dulbecco's modified Eagle's medium supplemented with 10% fetal calf serum, penicillin (100 U/ml) and streptomycin (50 µg/ml) and incubated at 37°C in a 5% CO_2 atmosphere. Human SH-SY5Y neuroblastoma cells (CRL-2266, ATCC) were cultured following manufacturer's instructions.

We used γ-secretase inhibitors DFK-167 (Millipore) at 10µM, and DAPT (N-[N-(3,5-Difluorophenacetyl)-L-alanyl]-S-phenylglycine t-butyl ester (Sigma, Aldrich) at 5µM for 20h. For mitophagy flux analysis, cells were treated with 1µM Antimycin A (Sigma,Aldrich) and 10µM Oligomycin A (Sigma,Aldrich) for 20h.

Quantitative real-time PCR

Total RNA was isolated using NucleoSpin RNA II (Macherey-Nagel) according to the manufacturer's protocol. Total RNA was extracted from cells using RNAeasy lipid tissue (Qiagen) according to the manufacturer's protocol. Complementary DNA (cDNA) was synthesized from 2 µg of total RNA and random primers using GoScript Reverse Transcription System kit (Promega). Target gene expression was analyzed by real time PCR using Corbett Rotor-Gene 6000 (Invitrogen) and SYBR Green (Roche Applied Sciences). Cycling parameters were as follows: 20 sec at 95 °C, 20 sec at 60 °C, and 20 sec at 72 °C for 55 cycles. Primer sequences: Drp1 (Forward 5'-TGGGCGCCGACATCA-3', Reverse 5'-GCTCTGCGTTCCCACTACGA-3'); Mfn1 (Forward 5'-GGCATCTGTGGCCGAGTT-3', Reverse 5'-ATTATGCTAAGTCTCCGCTCCAA-3'); and Mfn2 (Forward 5'-GCTCGGAGGCACATGAAAGT-3', Reverse 5'-ATCACGGTGCTCTTCCCATT-3'). Human Topoisomerase 1(Forward 5'-CCCTGTACTTCATCGACAAGG-3', Reverse 5'-CCACAGTGTCCGCTGTTTC-3'), and human GAPDH (Forward 5'-AGCCACATCGCTCAGACAC-3'; Reverse 5'-GCCCAATACGACCAAATCC-3') were used as control gene.

Imaging analyzes.

For the analysis of the mitochondrial three-dimensional network, after loading with 10 nM Mitotracker red KRB/1 mM $CaCl_2$ at 37°C for 30 min, cells were placed in a thermostated chamber on the stage of a Leica SP5 confocal microscope (Leica Microsystemes SA, France). Z-images were acquired with 63x objective, Mitochondrial objects number and volume were determined using 3D object Counter plugging [1].

Supplementary figure legends

Supplementary Figure 1 a mRNA levels (means ± SEM, n=4) of DRP1, MFN1 and MFN2 in SH-SY5Y stably expressing pcDNA3.1 empty vector (control) and APPswe cDNA (APPswe). **b-e** SDS-PAGE (b, c) and quantitative graphs (d, e) of the means ± SEM of MFN2, MFN1, and DRP1 expression levels in total cell extracts (n=4) (b, d) and mitochondrial-enriched fraction (n=5 in duplicates) (c, d) of control and APPswe cells. **f** Levels of complexes II-SDHB, III-UQCRC2, IV-COXII, and V-ATP5A subunits expression revealed using OXPHOS antibodies mix (see representative SDS-PAGE in Figure 1) and expressed as means ± SEM (n=4) control (taken as 100%). **g-k** Spectrophotometric analysis of the respiratory chain complex II, III, IV, and V activities are expressed as absolute values in nanomols of substrate/min/mg of proteins and presented as means ± SEM (n=3). Citrate synthase activity was analyzed to ascertain similar mitochondria content between control and APPswe expressing cells. **a, d-k** ** P <0.01, *** P <0.001, and ns: non-significant versus control using Mann Whitney test.

Supplementary Figure 2 a Representative images of mitochondrial structure in SH-SY5Y cells expressing APPswe treated with vehicle or with γ-secretase inhibitor (γ-sec inh) (5µM, 20h). Scale bars correspond to 10µm. **b, c** Quantitative analyses of objects number (b), and volume (voxels) (c) expressed as means ± SEM (n=3). **d** mRNA levels (means ± SEM of four independent experiments) of DRP1, and MFN2 in APPswe treated as in (a). **e** SDS-PAGE and quantification (means ± SEM, n=4) of mitochondrial MFN2, and DRP1 in APPswe treated as in (a). **b-e** * P <0.01, and ns: non-significant versus vehicle-treated APPswe cells using Mann Whitney test.

Supplementary Figure 3 a SDS-PAGE (low and high exposures) of APP and its indicated metabolites (revealed as in Figure 2) in vehicle (-)- or β-secretase inhibitor- (β-sec inh) (+) (30µM, 20h) treated APPswe cells revealed in total cell extracts (Tot), and mitochondrial-enriched fraction (Mit). Actin was used as loading control. **b, c** Quantitative graphs of indicated proteins in total extracts (b) and mitochondrial fraction (c) expressed as means ±

SEM (n=4) versus vehicle-treated APPswe cells (taken as 100%). **d** Electron microscopy ultrastructure of SH-SY5Y APPswe treated as in (a). Scale bars correspond to 2µm. N: nucleus. Colored arrowheads indicate mitochondria classes as shown in Fig. 1b. **e** Quantitative graph of mitochondria classes obtained in duplicates (> 10 different fields, > 150 mitochondria). **f** SDS-PAGE and quantification of NDUFB8 expression (means ± SEM, n=4) of vehicle-treated APPswe cells (taken as 100%). **g** Spectrophotometric analysis of the respiratory chain complex I activity expressed as absolute values in nanomols of substrate/min/mg of proteins and presented as means ± SEM, n=4. **h** TMRM median intensity obtained by FACS analyses. Data are expressed as means ± SEM, n=5 of vehicle-treated APPswe cells (taken as 100%). **i** Caspase 3-like activity under staurosporin stimulation (1µM, 2h) expressed as fluorescence intensity (Fluorescence units (FU)/mg protein/h) ± SEM, n=5 in triplicates. **j** SDS-PAGE of LC3-I and LC3-II, SQSTM1/p62 (p62), Parkin, PINK1, TOMM20, TIMM23, and HSP10 in mitochondria-enriched fraction of APPswe cells treated as in (a). Tubulin was used as loading control. **b, c** *** *P* <0.001, and ns: non-significant using Kruskal-Wallis test and Dunn's multiple comparison post-test. **g-i** ** *P* <0.01, versus vehicle-treated APPswe cells using *t* test.

Supplementary Figure 4 a SDS-PAGE (low and high exposures) of APP, C99, and C83 detected using APP-Cter antibody in SH-SY5Y cells stably expressing pcDNA3.1 empty vector (control) and APPswe construct (APPswe) in total cell extracts (Tot) and mitochondrial-enriched fraction (Mit). **b** SDS-PAGE and the quantification (means ± SEM, n=4) of APP, C99, and C83 detected as in (a) in SH-SY5Y cells expressing pcDNA3.1 (control) treated with vehicle (-) or with γ-secretase inhibitor (+) (γ-sec inh) (5µM, 20h). **c** Immunostaining of SH-SY5Y control cells treated as in (b) with APP-Cter (green) in combination with anti-HSP60 (red). Nuclei were labeled with DAPI. Images show merge signals reflecting the colocalization in yellow. Scale bars represent 10µm. **d** Electron microscopy ultrastructure of SH-SY5Y control cells treated as in (b). Scale bars correspond to 2µm.

N: nucleus. Colored arrowheads indicate mitochondria classes as shown in Fig. 1b. **e-h** Quantitative graphs of mitochondria classes (e) and the means ± SEM of mitochondria area (μm^2) (f), perimeter (µm) (g), and number/10μm^2 (h). (d-h) Data were obtained in two independent experiments in duplicates. The quantification was done in at least 20 different fields (> 150 mitochondria). **b, f-h** * *P* <0.05, ** *P* <0.01, **** *P* <0.0001 using Kruskal-Wallis test and Dunn's multiple comparison post-test.

Supplementary Figure 5 a Protein levels of complexes II-SDHB, III-UQCRC2, IV-COXII, and V-ATP5A in APPswe treated with vehicle (-) or with γ-secretase inhibitor (γ-sec inh) (+) (5µM, 20h) (see representative SDS-PAGE in Figure 3). Means ± SEM, n=4 versus control (taken as 100%). **b-f** Spectrophotometric analysis of the respiratory chain complexes II, III, IV, and V activities in APPswe treated as in (a) expressed as absolute values nanomols of substrate/min/mg of proteins and presented as means ± SEM, n=3. Citrate synthase activity was analyzed to ascertain similar mitochondria content between samples. **g, h** Representative plots of FACS analyses of mitochondrial potential (TMRM intensity plotted against cells counts) (g) and ROS (MitoSox intensity plotted against FSCH (forward light scatter)) (h) in SH-SY5Y cells stably transfected with pcDNA3.1 empty vector (control) or APPswe cDNA (APPswe) treated as in (a). P1 and P2 gates represent low and high TMRM intensity respectively (g). **i** Caspase 3-like activity in APPswe expressing cells under basal and STS stimulation (1µM, 2h) upon treatment with vehicle, DFK (10µM, 20h) or DAPT (5µM, 20h). The graph represents fluorescence intensity (Fluorescence units (FU)/mg protein/h) ± SEM, n=4. **a-f** ns: non-significant versus control using Mann Whitney test. **i** **** *P* <0.0001 using one-way ANOVA and Tukey's multiple comparison post-test.

Supplementary Figure 6 a-d TMRM (a, b) and MitoSox (c, d) median intensities obtained by FACS analyses in SH-SY5Y cells stably transfected with pcDNA3.1 empty vector (control) (a, c) or in MEF WT and MEF APPKO (b, d) treated with vehicle (-) or γ-secretase inhibitor (γ-sec inh) (+) (5µM, 20h). Data are presented as means % ± SEM, n=3-5 of controls (taken as 100%). **e** Caspase 3-like activity was assessed under basal and STS stimulation (1µM, 2h) in the same experimental conditions

described as in (b, and d). Fluorescence intensities expressed in Fluorescence units (FU)/mg protein/h and are the means ± SEM, n=4. **a-d** * P <0.05, ns: non-significant versus control using Mann Whitney test. **e** **** P <0.0001, and ns: non-significant using one-way ANOVA and Tukey's multiple comparison post-test.

Supplementary Figure 7 a Expression levels of complexes II-SDHB, III-UQCRC2, IV-COXII, and V-ATP5A in HEK cells transiently transfected with pcDNA3.1 empty (control) or C99 construct (see representative SDS-PAGE in Figure 4). Means ± SEM, n=5 are presented versus control (taken as 100%). **b-f** Spectrophotometric analysis of the respiratory chain complex II, III, IV, and V activities expressed as absolute values in nanomols of substrate/min/mg of proteins and presented as means ± SEM, n=4. Citrate synthase activity was analyzed to ascertain similar mitochondria content between samples. **a-f** ns: non-significant versus control using Mann Whitney test.

Supplementary Figure 8 a Cells undergoing mitophagy (cells with fragmented red mitochondria revealed with Cox8-EGFP-mCherry probe) in APPswe cells treated with vehicle, oligomycin A (OA) (10µM, 20h), or with OA and antimycin A (AA) (1µM, 20h) presented as means ± SEM, n>100 cells. **b** Immunostaining of APPswe cells treated with vehicle or with γ-secretase inhibitor (γ-sec inh) (5µM, 20h) and stained with p62, Parkin or phospho-poly-ubiquitin (p-S65-Ub: p-Ub) antibodies (green) and anti-HSP60 (red). **c** Representative images of APPswe cells transfected with LAMP1-GFP probe (green) and Mit-RFP probe (red) and treated as in (b). The graph shows the colocalization (Mander's coefficient) of Mit-RFP with LAMP1-GFP, means ± SEM of n=14 cells. **d** Immunostaining of SH-SY5Y cells stably expressing control and C99 constructs and treated with vehicle or with γ-secretase inhibitor (γ-sec inh) (5µM, 20h) and stained with anti-Parkin (green) and anti-HSP60 (red). **b-d** Images show merge of green and red signals reflecting the colocalization in yellow. Scale bars represent 10µm. Where indicated, nuclei were labeled with DAPI. **a** ** P <0.01, and ns: non-significant using one-way ANOVA and Tukey's multiple comparison post-test.**c** ns: non-significant versus vehicle using Mann Whitney test.

Supplementary Figure 9 a SDS-PAGE of LC3-I and LC3-II, SQSTM1/p62 (p62), Parkin, TIMM23, and HSP10 in mitochondria-enriched fraction of SH-SY5Y control cells treated with vehicle (-) or γ-secretase inhibitor (γ-sec inh) (+) (5µM, 20h). Tubulin was used as loading control. **b-c** Expression levels of indicated proteins as means ± SEM, n=3-5 versus control (taken as 100%). **d** Immunostaining of SH-SY5Y control treated as in (a) and stained with p62, Parkin or phospho-poly-ubiquitin (p-S65-Ub: p-Ub) antibodies (green) and anti-HSP60 (red). **e** Representative images SH-SY5Y cells transfected with LAMP1-GFP probe (green) and Mit-RFP probe (red) and treated as in (a). The graph shows the colocalization (Mander's coefficient) of Mit-RFP with LAMP1-GFP, means ± SEM of n=14 cells. **d, e** Images show merge of green and red signals reflecting the colocalization in yellow. Scale bars represent 10µm. Nuclei were labeled with DAPI. **b, c** * *P* <0.05, ** *P* <0.01, **** *P* <0.0001, and ns: non-significant using Mann Whitney test.

Supplementary Figure 10 a Brain section of 12 months old AAV-C99 injected mice stained with APP-Cter antibody (shown also in Figure 8a). Boxed area represents subiculum region analyzed by electron microscopy. Scale bar represent 500µm. **b** Electron microphotographs of neuronal soma of young (2-3 months old) AAV-C99-injected mice. N: nucleus. Yellow and red arrows indicate mitochondria classes I or II shown in representative images in (c). **c-e** Quantitative graphs of mitochondria classes (c) and of the means ± SEM of mitochondria area (μm^2) (d), and perimeter (µm) (e). (b-e) Data were obtained in two mice for each condition (> 20 analyzed field, >100 of measured mitochondria area and perimeter). **c** * *P* <0.05 using Kruskal-Wallis test and Dunn's multiple comparison post-test. **d, e** ns: non-significant versus respective AAV-free using Mann Whitney test.

Supplementary Figure 11 a-d Correlation plots between Aβ and LC3-II/I (a), p62 (b), Parkin (c), and PINK1 (d) levels in controls (n=6-7, white dots), and AD (n=9-10, red dots) brains. **e** SDS-PAGE and quantification of pTau detected with AT8 antibody. Graph represents mean ± SEM versus control taken as 100% of control (n=8) and AD brains (n=13) (patients information in suppl. Table 1). **f-i**

Correlation plots between pTau and LC3-II/I (f), p62 (g), Parkin (h), and PINK1 (i) levels in controls (n=6-7, white dots), and AD (9-10, red dots) brains. **a-d, f-i** Linear regression was used to determine *P* and goodness of fit (R^2) values.

References

1. Cordelieres FP, Bolte S (2014) Experimenters' guide to colocalization studies: finding a way through indicators and quantifiers, in practice. Methods Cell Biol 123:395-408. doi:10.1016/B978-0-12-420138-5.00021-5
2. Heber S, Herms J, Gajic V, Hainfellner J, Aguzzi A, Rulicke T, von Kretzschmar H, von Koch C, Sisodia S, Tremml P, Lipp HP, Wolfer DP, Muller U (2000) Mice with combined gene knock-outs reveal essential and partially redundant functions of amyloid precursor protein family members. J Neurosci 20:7951-7963

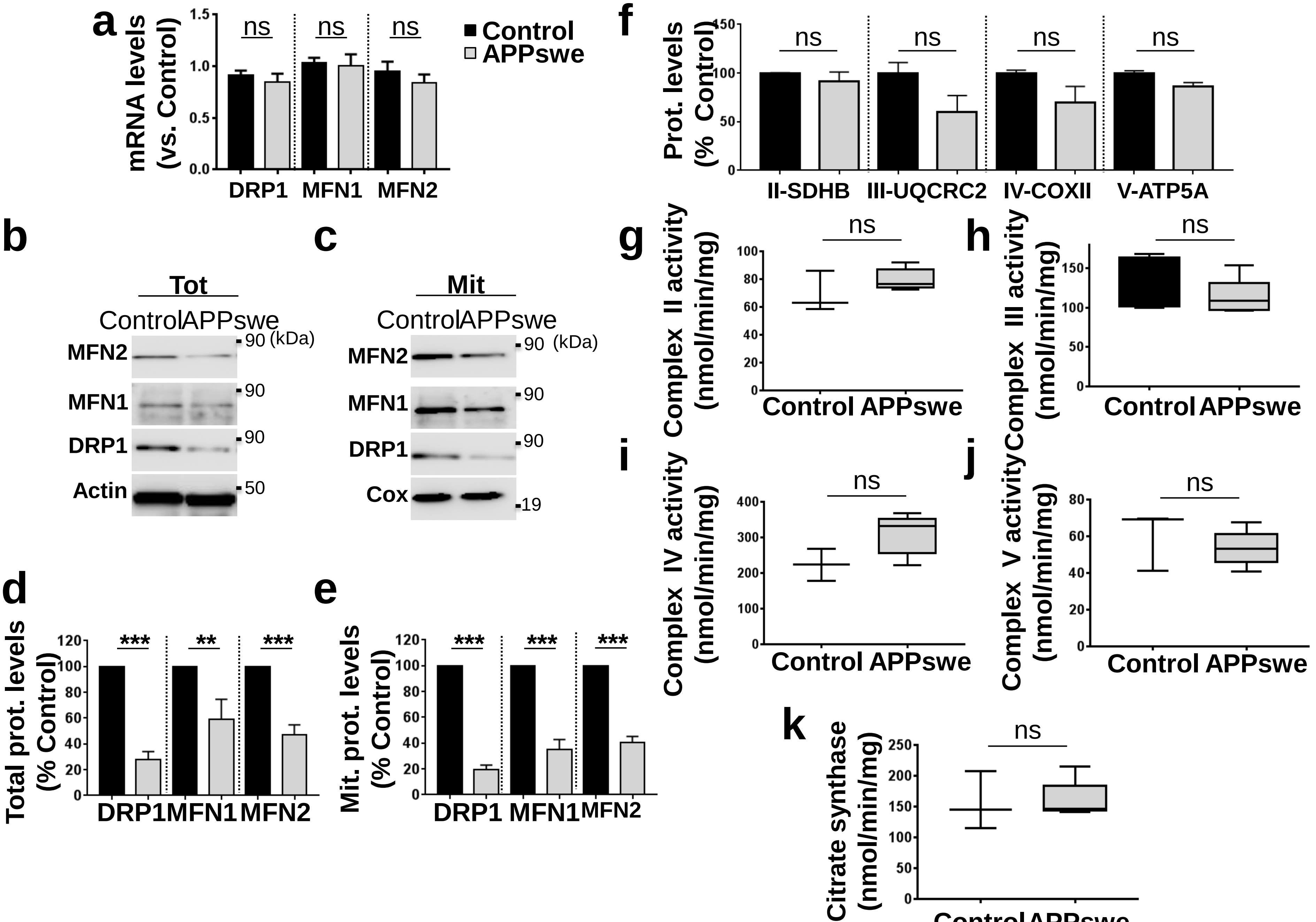

Suppl. Figure 1

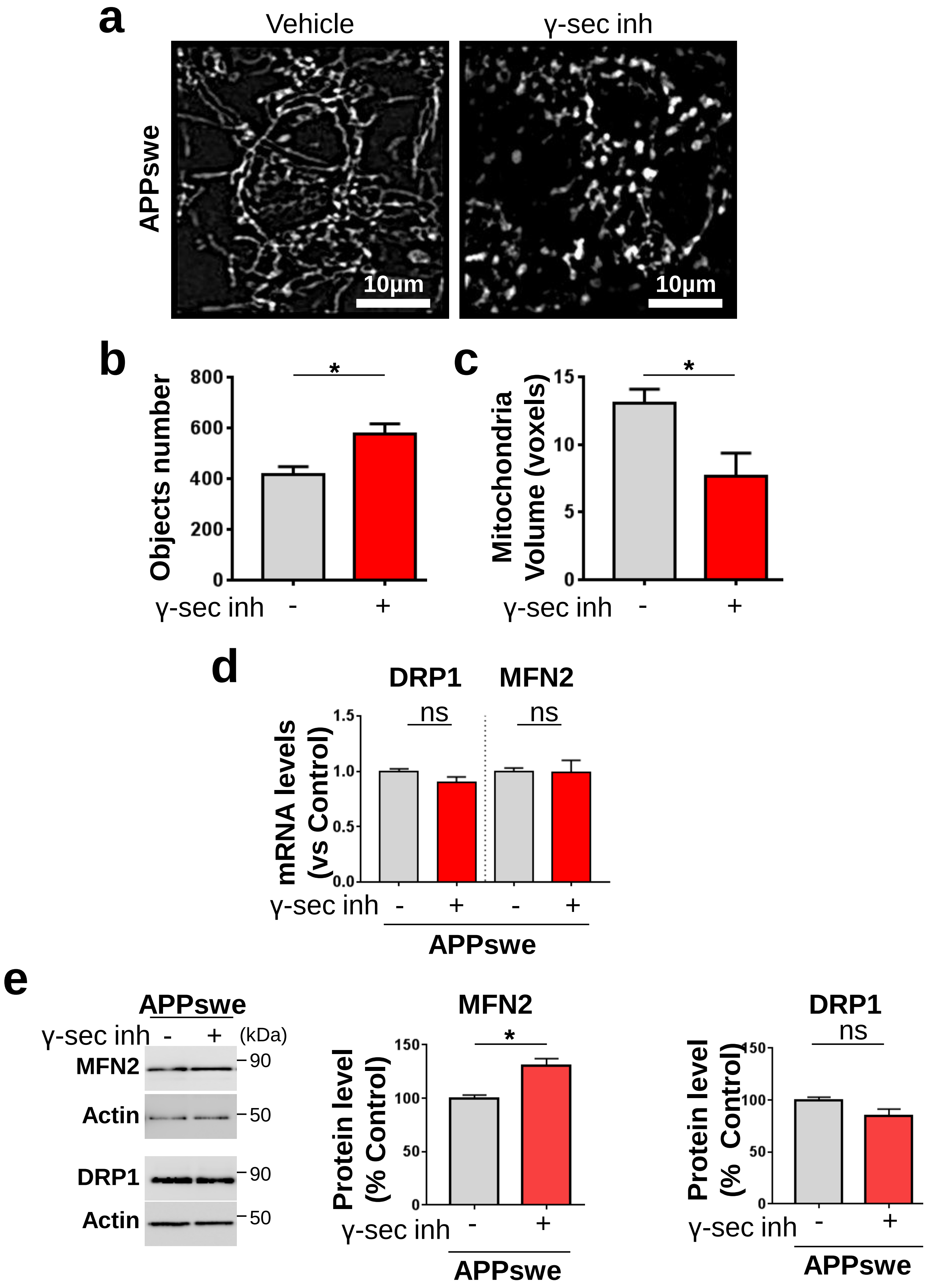

Suppl. Figure 2

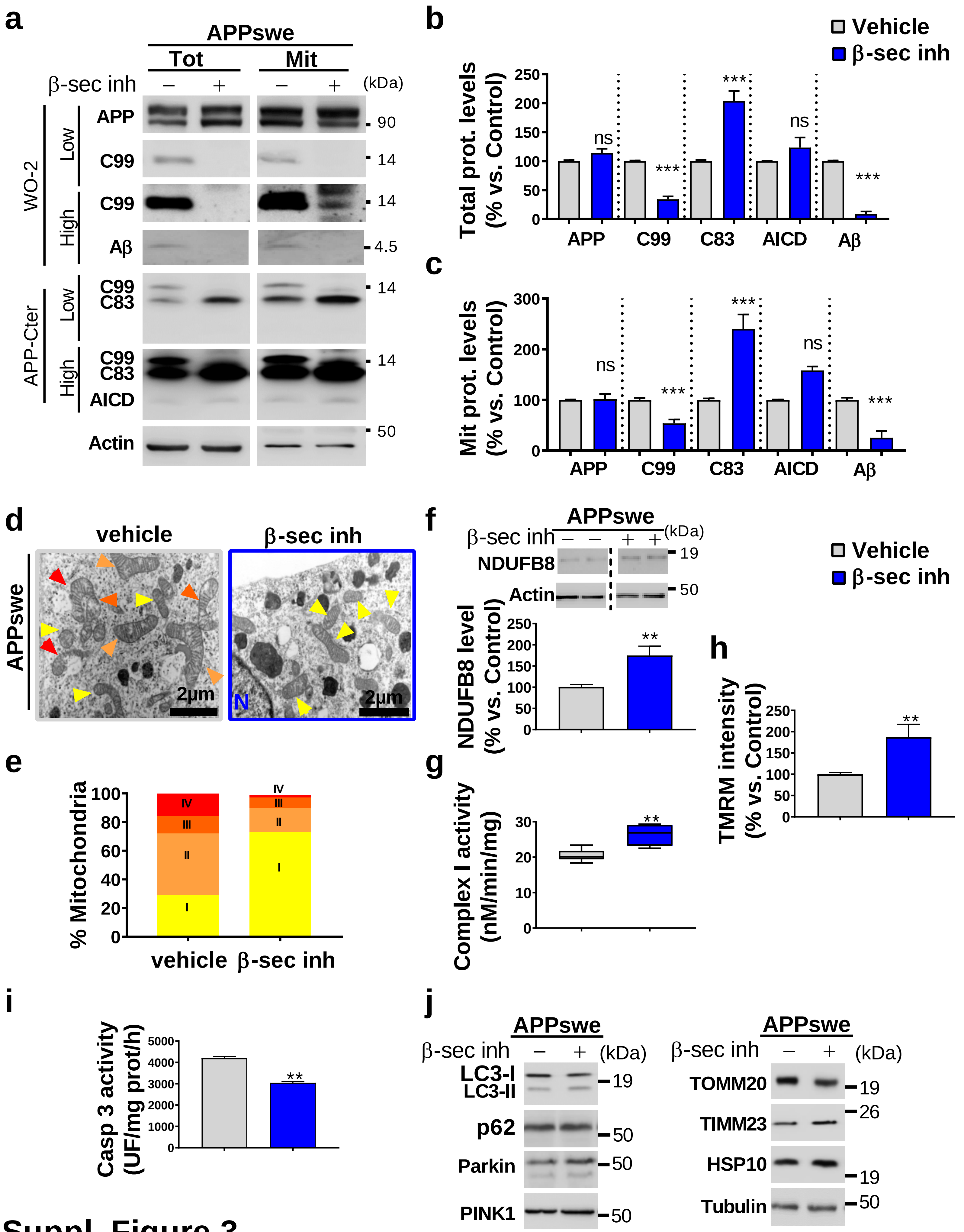

Suppl. Figure 3

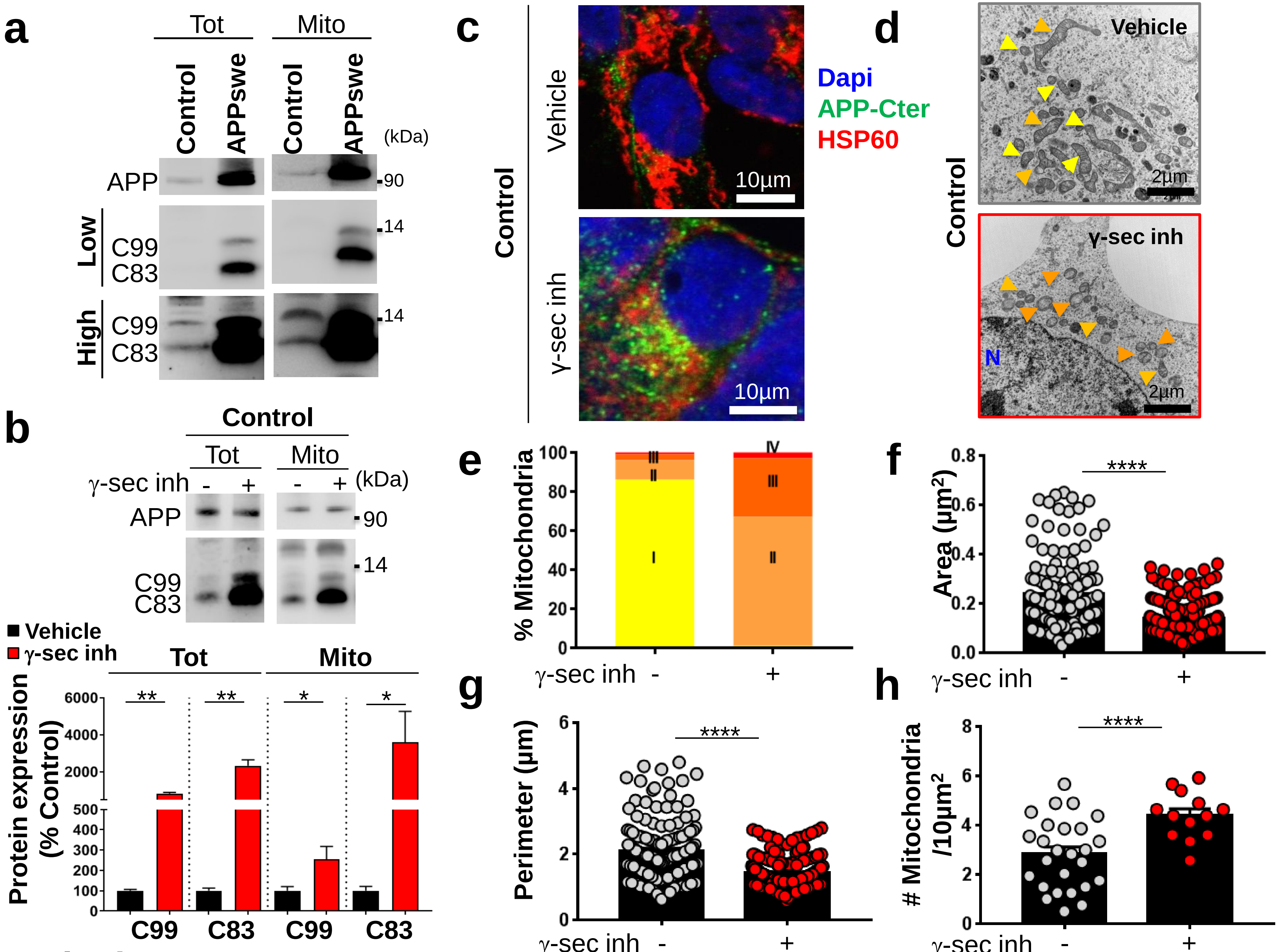

Suppl. Figure 4

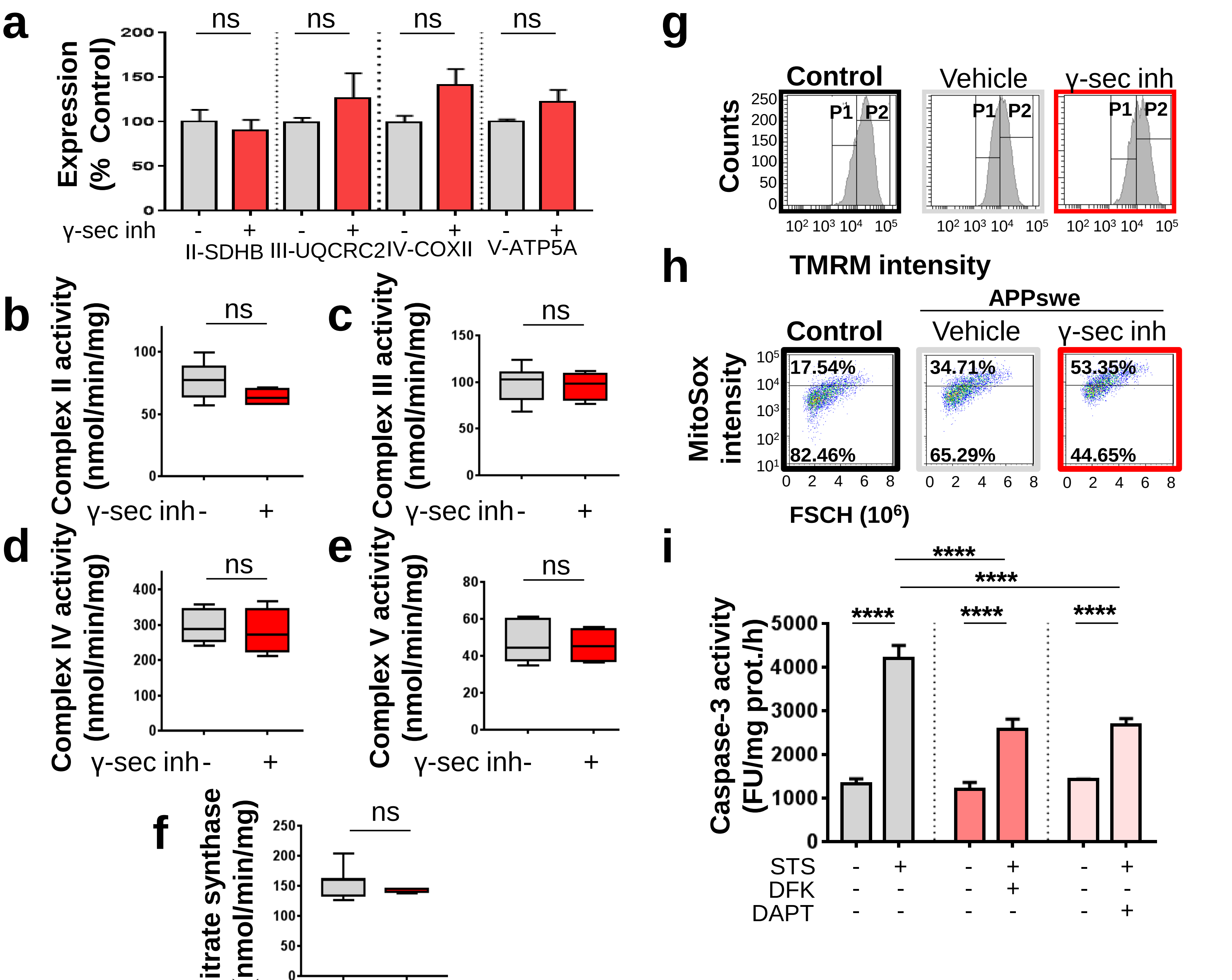

Suppl. Figure 5

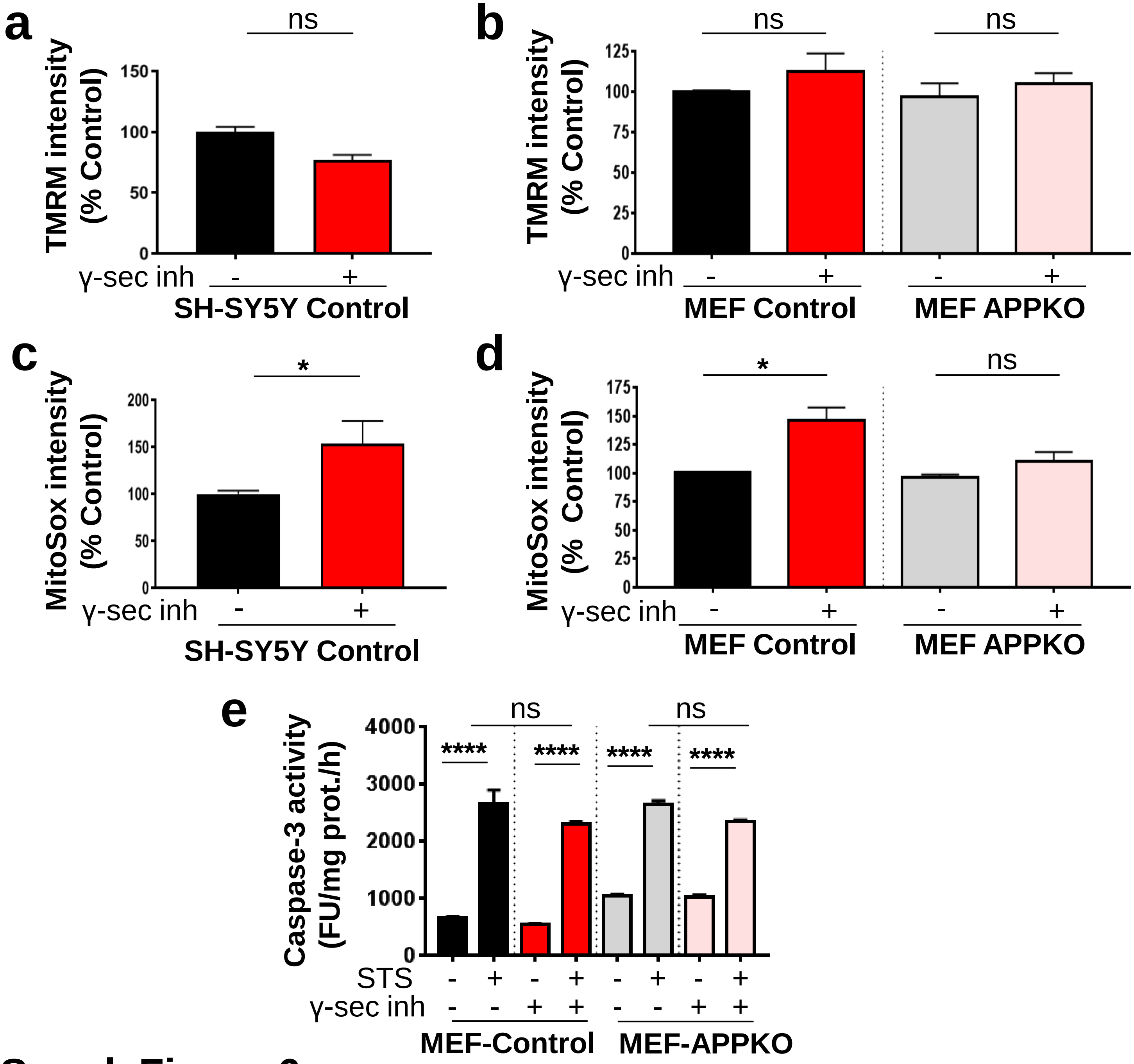

Suppl. Figure 6

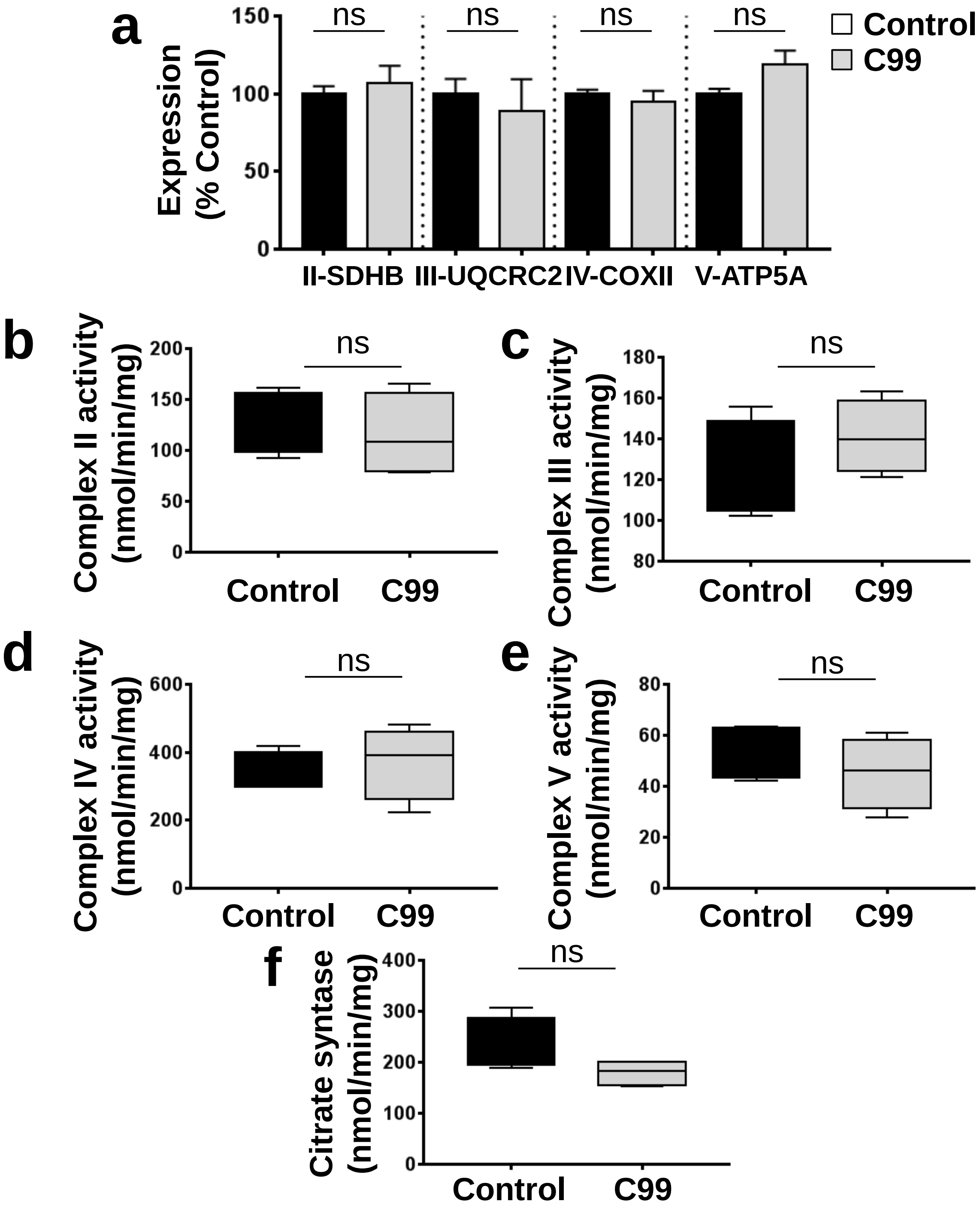

Suppl. Figure 7

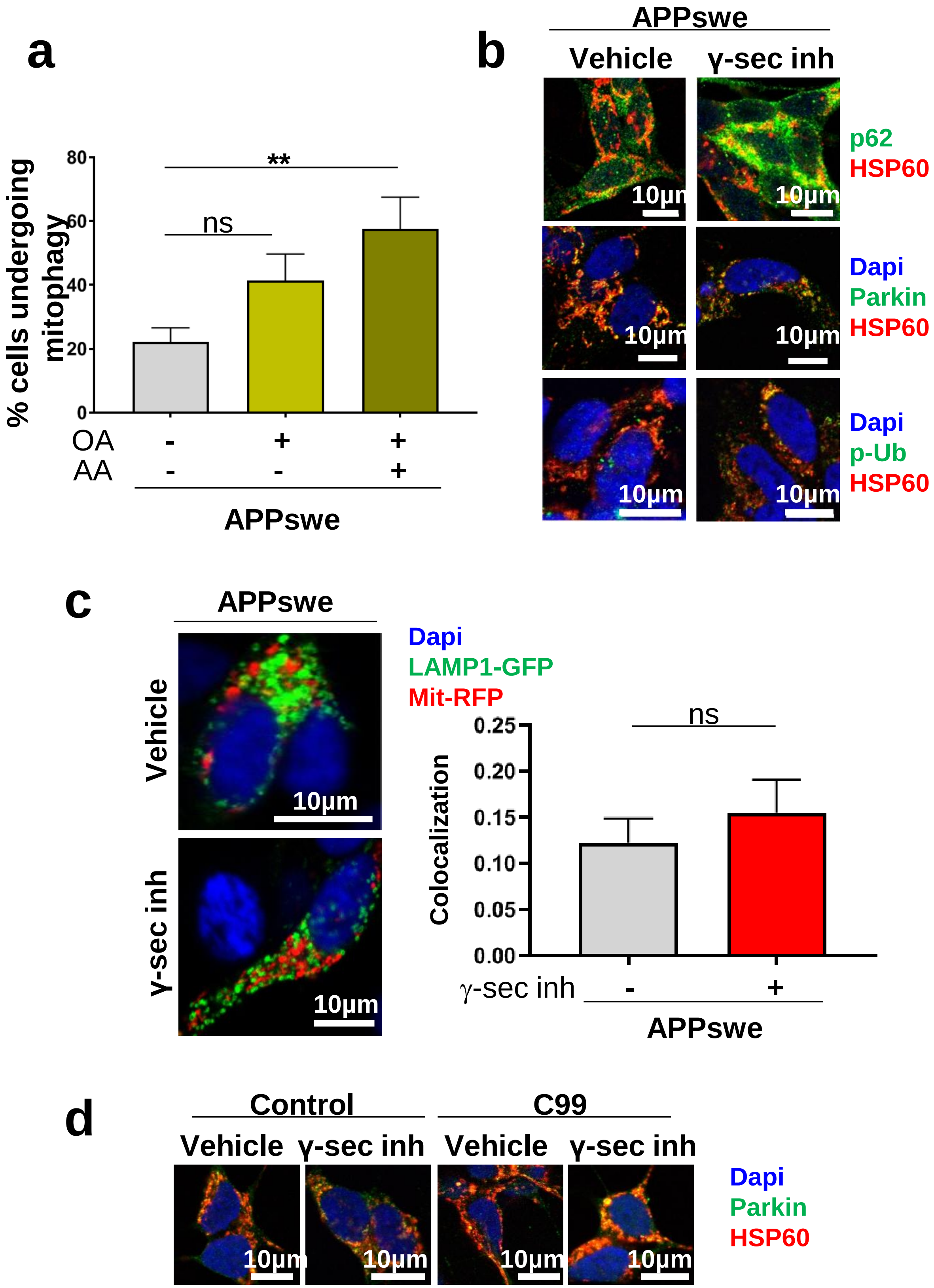

Suppl. Figure 8

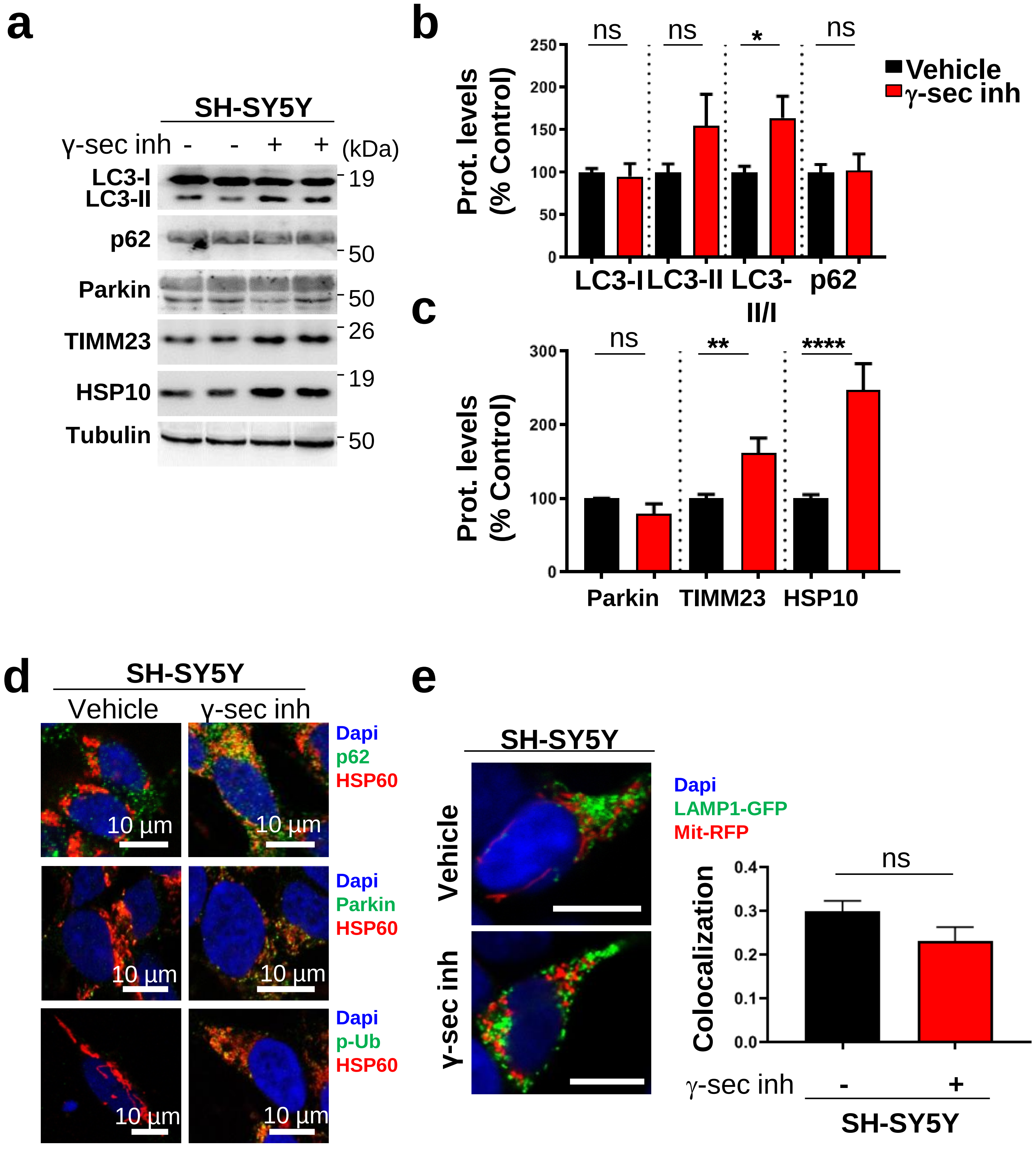

Suppl. Figure 9

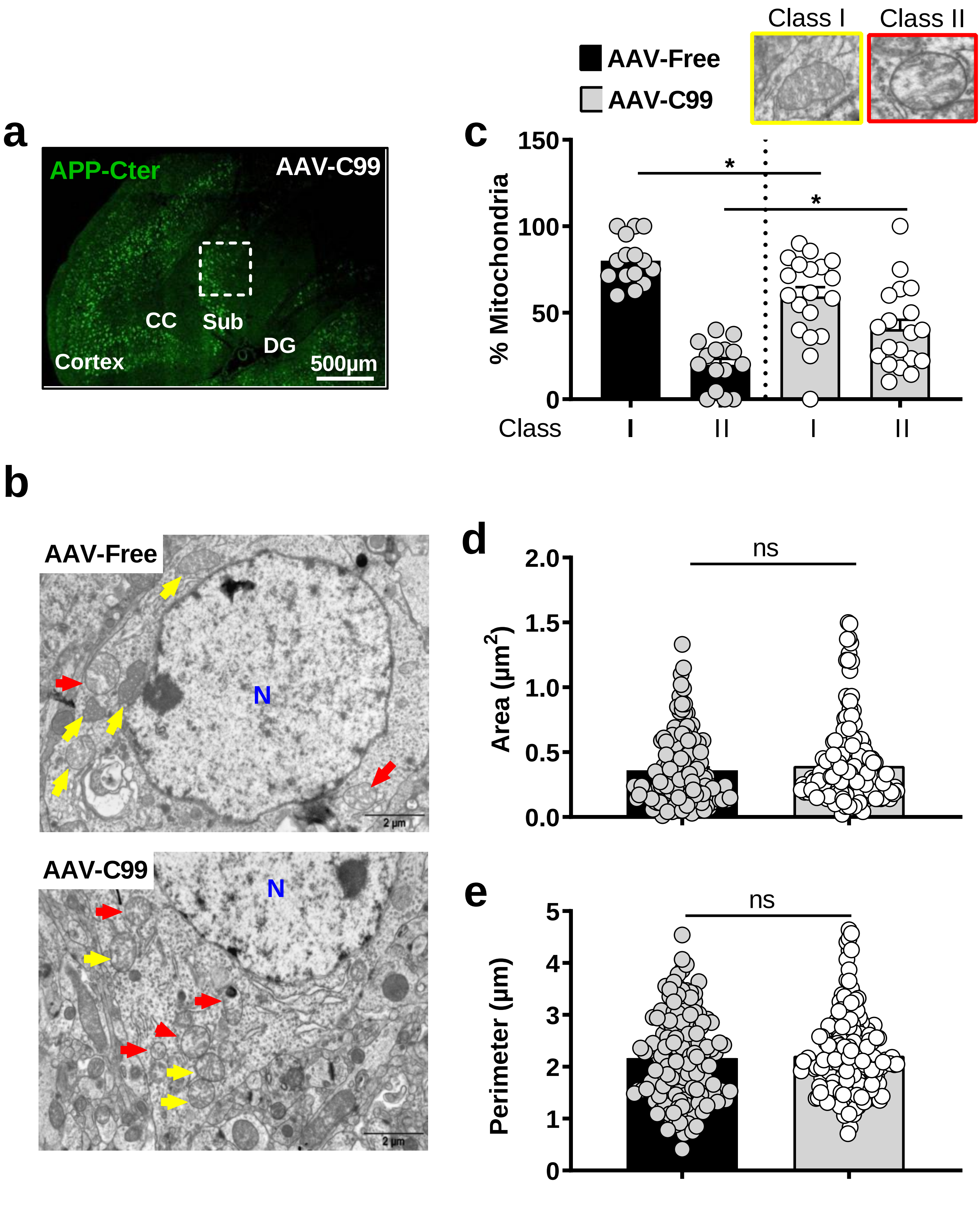

Suppl. Figure 10

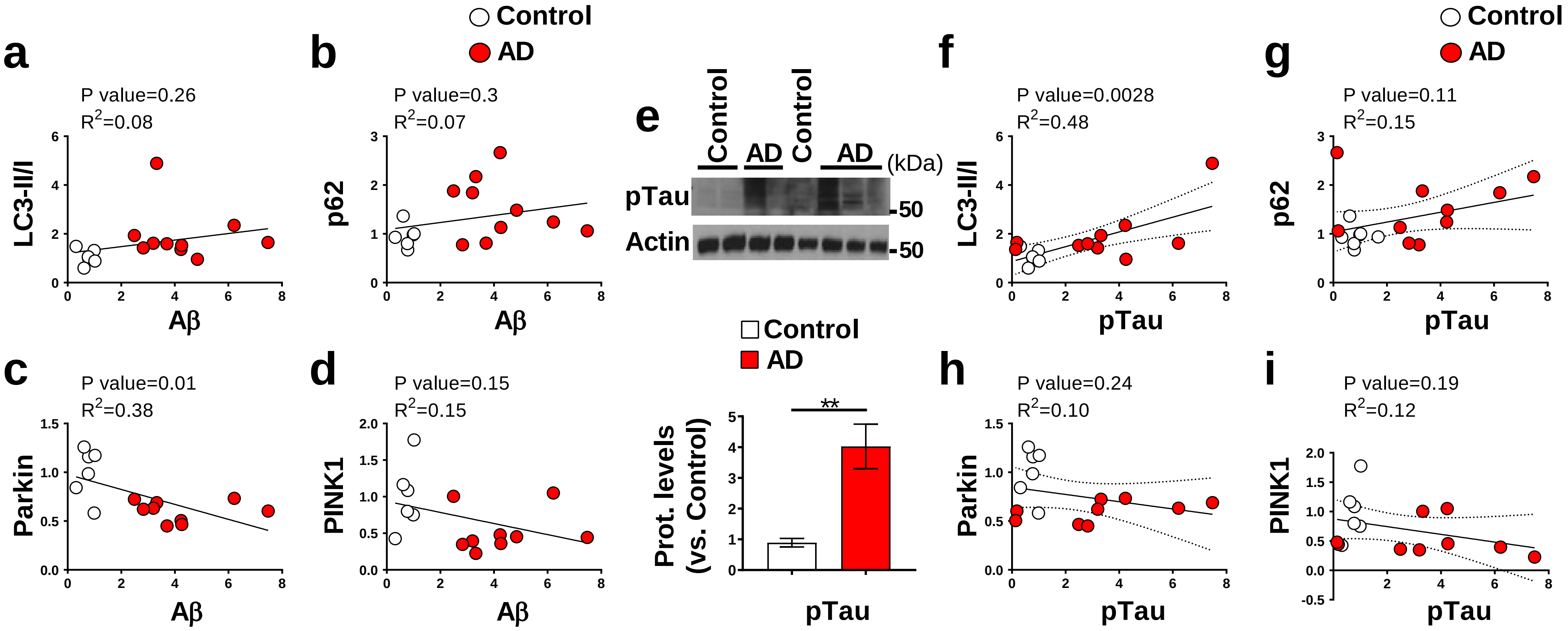

Suppl. Figure 11

Table 1: Demographic data and neuropathological findings related to human brain samples used in SDS-PAGE analyses (temporal lobe), and brain-derived slices used in immunohistochemistry analyses (T1 region of the temporal lobe)

	**Age (years)**	**Gender**	**PMD (h)**	**Braak's NFT stage#**
Brain samples used in SDS-PAGE analyses				
Control	71	Male	26	-
Control	84	Male	32	-
Control	71	Female	15	-
Control	55	Male	25	-
Control	78	Male	35	-
Control	61	Male	20	-
ALS	62	Female	44	-
ALS	55	Male	21	-
ALS	62	Female	21	-
AD	68	Female	51	I-II
AD	68	Female	10	I-II
AD	80	Female	51	V
AD	84	Female	81	V
AD	65	Female	41	VI
AD	81	Male	19	VI
AD	89	Female	26	VI
AD	93	Female	21	VI
AD	91	Female	34	VI
AD	55	Female	58	VI
AD	79	Male	31	VI
AD	86	Male	32	VI
AD	75	Female	7	VI
AD	81	Female	NA	VI
AD	82	Female	NA	VI
Brain-derived slices used in immunohistochemistry analyses				
AD	81	Female	NA	VI
AD	55	Female	NA	IV
AD	79	Male	NA	VI
AD	84	Female	NA	V
AD	65	Female	NA	V
AD	80	Female	NA	V

PMD: Post mortem delay, h: hours, NA: not available. Controls are brain samples isolated from post-mortem patients diagnosed as negative for several neuropathologies. ALS are control brains samples diagnosed as negative for AD pathology obtained from pos-mortem patients diagnosed with Amyotrophic lateral sclerosis. # Braak and Braak's NFT (neurofibrillary tangle: tau-related pathology) stage, (-) means no NFT detection

Supplementary Table 2: List of antibodies used in the study

**Antibody**	**Host**	**Supplier**	**WB dilution**	**IF dilution**
6E10 (1-16 aa of Aβ) (SIG-39320)	Mouse	Covance/SIGMA	1/1000	1/2000
WO-2 (4-10 aa of Aβ) (MABN10)	Mouse	SIGMA	1/1000	-
APP Cter (A8717)	Rabbit	Gift from P. Fraser (Toronto)	1/1000	1/5000
82E1 (1st free aa of Aβ and C99) (10323)	Mouse	IBL America	1/1000	1/1000
pTau (Ser202, Thr205) (AT8)	Mouse	ThermoFisher	1/1000	-
HSP60 (H-1; sc-13115)	Mouse	Santa Cruz Biotechnologies	1/1000	1/200
HSP60 (N-20; sc-1052)	Goat	Santa Cruz Biotechnologies	1/2000	1/200
HSP10/Cpn10 (ADI-SPA-110)	Rabbit	StressGen	1/1000	-
p62/SQSTM1 (NBP1-49956)	Rabbit	Novus Biologicals	1/1000	1/2000
Parkin (MAB5512)	Mouse	Millipore	1/1000	1/500
TIMM23 (611222)	Mouse	BD Transduction Laboratories	1/5000	1/200
TOMM20 (612278)	Mouse	BD Transduction Laboratories	1/5000	-
P-Ub pS65 (A110)	Rabbit	Boston Biochem	1/1000	1/200
Pink1 (C2; AC-R3173-2)	Rabbit	Abiocode	1/1000	-
Cleaved Caspase 3 (Asp175)	Rabbit	Cell Signaling Technology	1/1000	-
Human OXPHOS (110411)	Mouse	Abcam	1/1000	-
LC3/ MAP1LC3B (NB100-2220)	Rabbit	Novus Biologicals	1/1000	-
Mitofusin 1 (ARP57702)	Rabbit	Aviva System Biology	1/1000	-
Mitofusin 2 (ab56889-100)	Mouse	Abcam	1/1000	-
Drp1/DLP1 (611112)	Mouse	BD Transduction Laboratories	1/1000	-
Cox VI (3E11)	Rabbit	Cell signaling	1/5000	-
Tubulin	Mouse	Sigma Aldrich	1/2000	-
β-Actin (AC-74)	Mouse	Sigma Aldrich	1/5000	-

Supplementary Tables 3: Mitochondria size and morphology in mice (a) and cellular models models (b). Statistical values are depicted in corresponding graphs.

(a) Mice models	Brain region	Area (μm^2)	Perimeter (µm)	Mitochondria Classe I (%)	Mitochondria Class II (%)
WT (5mo)	Subiculum	0.35 ± 0.013	2.24 ± 0.044	91 ± 2	9 ± 2
WT (17mo)	Subiculum	0.23 ± 0.009	1.8 ± 0.042	50 ± 6	50 ± 6
WT (5mo) + γ-sec inh	Subiculum	0.38 ± 0.013	2.22 ± 0.062	52 ± 8	48 ± 8
3xTgAD (5mo)	Subiculum	0.27 ± 0.015	1.9 ± 0.062	79 ± 3	21 ± 3
3xTgAD (17 mo)	Subiculum	0.33 ± 0.012	2.12 ±0.051	18 ± 4	82 ± 4
2xTgAD (17 mo)	Subiculum	0.317 ± 0.014	2.1 ± 0.054	19 ± 4	81 ± 4
3xTgAD (5mo) + γ-sec inh	Subiculum	0.45 ± 0.03	2.4 ± 0.085	19 ± 5	81 ± 5
AAV-Free (2-3 mo)	Cortex	0.21 ± 0.017	1.72 ± 0.095	91 ± 3	9 ± 3
AAV-Free (12 mo)	Cortex	0.16 ± 0.011	1.5 ± 0.065	96 ± 2	4 ± 2
AAV-C99 (2-3 mo)	Cortex	0.22 ± 0.014	1.76 ± 0.07	47 ± 5	53 ± 5
AAV-C99 (12 mo)	Cortex	0.25 ± 0.011	2 ± 0.058	52 ± 6	48 ± 6
AAV-Free (2-3 mo)	Subiculum	0.35 ± 0.021	2.16 ± 0.07	80 ± 3.4	20 ± 3.5
AAV-C99 (2-3 mo)	Subiculum	0.34 ± 0.014	2.21 ± 0.053	60 ± 5.4	40 ± 5.4

(b) Cellular models	Area (μm^2)	Perimeter (µm)	Number/10μm^2	Mitochondria Classe I (%)	Mitochondria Class II (%)	Mitochondria Classe III (%)	Mitochondria Classe IV (%)
Control	0.41 ± 0.018	2.19 ± 0.07	2.8 ± 0.27	86 ± 3	10 ± 2	3 ± 2	1 ± 1
APPswe	0.8 ± 0.043	3.22 ± 0.10	1.8 ± 0.18	29 ± 5	43 ± 5	12 ± 3	16 ± 4
Control + γ-sec inh	0.26 ± 0.009	1.5 ± 0.028	4.39 ± 0.26	1 ± 1	66 ± 6	30± 6	3 ± 1
APPswe + γ-sec inh	0.47 ± 0.017	2.05± 0.045	3.5 ± 0.27	1 ± 1	47 ± 6	50 ± 6	2 ± 1
APPswe + β-sec inh	0.59 ± 0.03	3.4 ± 0.1	2.3 ± 0.3	76 ± 6	13 ± 5	7 ± 3	4 ± 2
